# Chloroplast Genome Diversity and Marker Potentials of Diverse *Ensete ventricosum* Accessions

**DOI:** 10.3390/ijms26199561

**Published:** 2025-09-30

**Authors:** Manosh Kumar Biswas, Bulbul Ahmed, Mohamed Hijri, Trude Schwarzacher, J. S. (Pat) Heslop-Harrison

**Affiliations:** 1 African Genome Center (AGC), University Mohamed VI Polytechnic, Lot 660, Hay Moulay Rachid, Benguerir BP43150, Morocco; bulbul.ahmed@um6p.ma (B.A.); mohamed.hijri@um6p.ma (M.H.); 2 Genetics and Genome Biology, University of Leicester, Leicester LE1 7RH, UK; ts32@leicester.ac.uk; 3 Institut de Recherche en Biologie Végétale, Département de Sciences Biologiques, Université de Montréal, 4101 Rue Sherbrooke Est, Montréal, QC H1X 2B2, Canada

**Keywords:** plastid genome, comparative genomics, phylogenomic, molecular marker, genomic resource, RNA editing

## Abstract

*Ensete ventricosum* is a morphologically gigantic, monocot, diploid sister to the banana plant species. It is commercially cultivated as a starch source, only in Ethiopia, where it feeds twenty million people. Here, the complete chloroplast (CP) genomes of 15 diverse landraces of *E. ventricosum* were assembled and annotated, for comparative genomics, genetic diversity analysis, and molecular marker development. The assembled *E. ventricosum* CP genomes ranged between 168,388 and 168,806 bp. The sampled CP genomes were quadripartite in structure and had two single-copy regions, a large single-copy region (LSC, average length 88,657 bp), and a small single-copy region (SSC, average length 11,098 bp) separated by inverted repeat regions (IR, average length 34,437 bp). The total number of annotated genes varies between 135 and 138, including 89–92 protein-coding genes, 38 tRNA genes, and 4 rRNA genes. All CP genes, including non-functional ones and intergenic regions, were transcribed with the transcriptome, covering almost 92% of the *E. ventricosum* CP genome. Codon usage, amino acid frequency, GC contents, and repeat nucleotides were similar among the 15 landraces. Mono- and tetranucleotide simple sequence repeats (SSRs) were found more frequently than other SSRs. An average of 71% of these SSRs were located in the LSC region, and the majority of the SSR motifs were composed of A/T nucleotides. A phylogenetic analysis of the 15 *Ensete* landraces indicated a common evolutionary origin, while the China sample was positioned separately, suggesting notable genetic differences. This study presents a comparative analysis of the chloroplast genomes of 15 *E. ventricosum* landraces, providing valuable insights into their genetic diversity and evolution. The identified SSR markers and conserved genomic features offer essential resources for future research and an improvement in *Ensete* conservation and breeding.

## 1. Introduction

*Ensete ventricosum* (Enset or Abyssinian banana; Musaceae), a perennial monocarpic monocot, belongs to the family Musaceae, and the genus is composed of seven species [1]. *E. ventricosum* is the only extensively cultivated species in the *Ensete* genus, although some are of horticultural interest. It is a staple crop grown in Ethiopia for the starch in its corm, pseudostem, and leaf bases. Like its sister genus *Musa* (banana), the crop is mostly vegetatively propagated, although wild germplasm is seed-propagated. It has a great economic and social impact on millions of Ethiopians [2]. Some of its cultivars have been used for traditional medicine [3]. It is speculated that the phytochemical composition greatly differs among the cultivated varieties. Consequently, it is crucial to accurately identify all the cultivated and wild varieties of Enset using both morphological and molecular data. Several studies have attempted to identify and characterize the cultivated and wild Enset cultivars using morphological [4,5] and molecular markers, such as AFLP [6], RAPD [7], ISSR [8], and SSR [9,10]. Barcoding markers such as plastid DNA (*matk*, *rpl16*, *trnL-trnF*) and the cp-ssr marker are still underutilized for the exploration of *Ensete* spp., and available molecular marker data, genetic diversity data, and population structure information about this species are still insufficient. Cp-SSR markers, either alone or in combination with nuclear-SSR, are widely used in population genetics and phylogenomic studies in plants. Evidence suggests that species-specific primers are more productive than universal primers in identifying polymorphic loci in chloroplast genomes [11]. Consequently, it will be worth developing barcode and cp-ssr primer sets to explore *Ensete* germplasm (cultivated and wild accessions) diversity and resolve the vernacular naming puzzle of the *Enset* accessions.

The genomes of chloroplasts (plastids; with ctDNA, cpDNA, or plastome) range from 120 to 160 kb in size and contain 110 to 130 distinct genes, encoding ~80 unique proteins, 30 tRNAs, and 4 rRNAs [12,13], and they are normally maternally inherited. Most chloroplast genomes have a quadripartite circular structure, typically containing two copies of inverted repeat regions (IR, length between 20 and 28 kb) separated by 16–27 kb small single-copy (SSC) and large single-copy (LSC, typically 80–90 kb) regions [14]. In comparing species, structural variation is found, including the expansion of or reduction in the IR region, the presence of large deletions or inversions, pseudogenization, and gene loss [15,16]. The low rates of nucleotide substitution, lack of recombination [17], conservation in gene content and order, and composition of large numbers of single-copy genes in the cp genome are widely used to resolve the phylogenetic relationship at different taxonomic levels [18,19]. Comparative plastomics shows micro-structural variations among cp genomes in terms of single-nucleotide polymorphisms (SNPs), SSRs, and insertions/deletions (indels), often with hotspots of variation [20,21]. While infrageneric or intraspecific variation levels in chloroplast DNA sequences are low [22], their presence is important to study as they give important information about phytogeography and important diversification events in species or subspecies, both in diploid species such as *E. ventricosum* (2n = 2x = 18) and more complex groups of species. Chloroplast genome data complements the study of nuclear genome diversity: in *Musa*, many of the x = 11 groups include diploids (2n = 2x = 22), triploids [23], and tetraploids (FHIA-17 accession no: GY0109; Source: https://www.crop-diversity.org/mgis/accession-search?, Accessed on 30 March 2023 ). The complete cp genomes of *Musa acuminata* [24,25], *Musa balbisiana* [26,27], *Musa itinerans* [28], and *Musa beccarii* [25] have been assembled and annotated from whole genome sequences, and the phylogenetic relation is estimated with other monocot species, allowing us to study the genetic variation and diversity at both the intra- and interspecies levels of the *Musa* clade.

In this study, we aimed to construct the complete chloroplast genomes of 15 *E. ventricosum* landraces using publicly available whole genome raw-read data. We then performed a comprehensive comparative and evolutionary analysis, estimating genome structure, gene content, gene order, and overall genome size. Additionally, we explored the distribution and location of tandem repeats, SSR polymorphisms, indels (insertions and deletions), and SNPs (single-nucleotide polymorphisms). A genomic resource was also created, featuring CP-SSR, CAPS-SNP, and indel markers, which will assist in placing new accessions into the context of their maternal lineage.

## 2. Results

### 2.1. Chloroplast Genome Assembly, Features, Annotation, and Comparative Genomics

**Figure 1:**
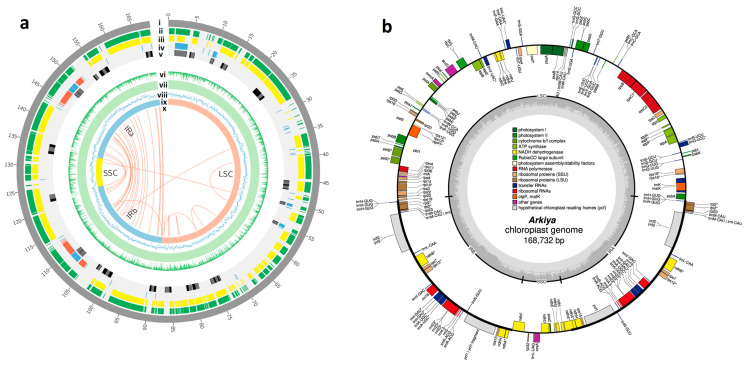
The assembly and annotation of the *Ensete ventricosum* chloroplast genome: (**a**) A Circos plot representing the assembly, genomic features, and annotations of the complete chloroplast genome of 15 accessions of *E. ventricosum*. [i] The length of the chloroplast genome is represented in kbps. [ii] The total genes annotated in the genome. [iii] Plotting only protein-coding genes. [iv] Plotting tRNA (sky color band) and rRNA (red color band) genes. [v] The black band represents exons, and the deep gray band represents introns. [vi] The sequence identity % of 15 landraces of *E. ventricosum* cp genomes against the reference (*Musa balbisiana*) chloroplast genome. [vii] The mapping coverage % of 15 landraces of *E. ventricosum* cp genomes against the reference (*M. balbisiana*) chloroplast genome. [viii] The average GC content % of 15 landraces of *E. ventricosum* cp genomes. [ix] Representing the 4 major regions of the cp genomes. [x] Representing the duplicate gene position on the *E. ventricosum* cp genomes. (**b**) A representative circular map of the *E. ventricosum* CP genome represents genes using colored boxes to denote their functional groups; genes with the boxes inside and outside the circle are transcribed in the clockwise direction and counterclockwise direction, respectively. The inner circle indicates the GC content and inverted repeat boundaries. * indicates that the gene contains an intron.

Complete chloroplast genomes were reconstructed from genomic DNA reads from 15 named landraces of *Ensete ventricosum* (Supplementary Figure S1 and Table S1). Gene order and genome organization were identical in all 15 assemblies (Figure S1a–n) although there was substantial variation between the landraces at the sequence level (genome size varied from 168,388 bp for ‘Chia’ to 168,806 bp for ‘Mazia’). The genome (Figure 1 and Figure S1) possesses the typical quadripartite structure with a pair of IR (inverted repeat) regions (34,334 to 34,523 bp) separated by an LSC (large single-copy) region (87,828 to 88,768 bp) and an SSC (small single-copy) region (11,040 to 11,487 bp), and it has a typical chloroplast GC content of 37%. The genomes encoded a total of 135 to 138 genes (among them, 82 to 85 unique genes) including 89 to 92 protein-coding, 37 tRNA, and 8 rRNA genes (Table 1 and Table S2).

*psbA* is the first gene in the LSC region with a total of 100 to 103 genes located in this region. SSC regions contain 9 to 10 genes, starting with *ndhF* genes. All the ‘ribosomal RNA’ (*rrn16*; *rrn23*; *rrn4.5*; *rrn5*) and ‘Conserved hypothetical chloroplast open reading frame’ (*ycf1*; *ycf2*) genes are only located in IR regions. Most tRNA genes are located in LSC regions, with a few in the IR regions and only one (*trnL-UAG*) in the SSC regions. All the ‘Large subunit of ribosomal protein’ (*rpl14*; *rpl16*; *rpl2*; *rpl20*; *rpl22*; *rpl23*; *rpl32*; *rpl33*; *rpl36*) genes are located in the LSC-IR junction and nearby regions. ‘Small subunit of ribosomal protein’ genes were distributed in LSC and IR regions, and they were not found in the SSC region with the remaining genes in the LSC region (Figure 1b and Figure S1a–n).

**Figure 2:**
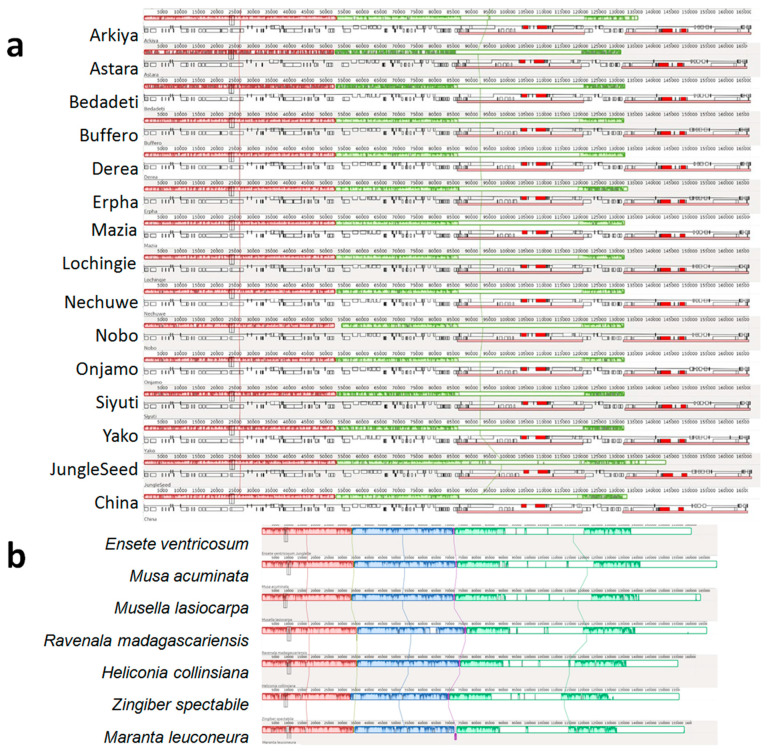
Mauve alignment: (**a**) shows the Mauve alignment of 15 CP genomes of diverse *E. ventricosum* landraces assembled in this study. (**b**) represents the Mauve alignment of *E. ventricosum* (landraces: JungleSeeds) with monocot relatives.

A comparative chloroplast genome alignment was performed using the Mauve tool to evaluate sequence conservation among the 15 *Ensete ventricosum* landraces (Figure 2a) and between *E. ventricosum* and related species within the Zingiberales order (Figure 2b). The results revealed minor structural variations and sequence divergence among *E. ventricosum* landraces. Additionally, species-specific insertions and deletions (indels) were detected, indicating potential genetic differentiation among landraces. Comparative alignment with other members of Zingiberales (Figure 2b) showed that *E. ventricosum* exhibits higher sequence similarity with *Musa acuminata* and *Musella lasiocarpa* compared to more distantly related species such as *Ravenala madagascariensis*, *Heliconia collinsiana*, *Zingiber spectabile*, and *Maranta leuconeura*. Notably, a small inversion was identified in the chloroplast genome of *Maranta leuconeura*, highlighting structural rearrangements in more distantly related taxa.

**Figure 3:**
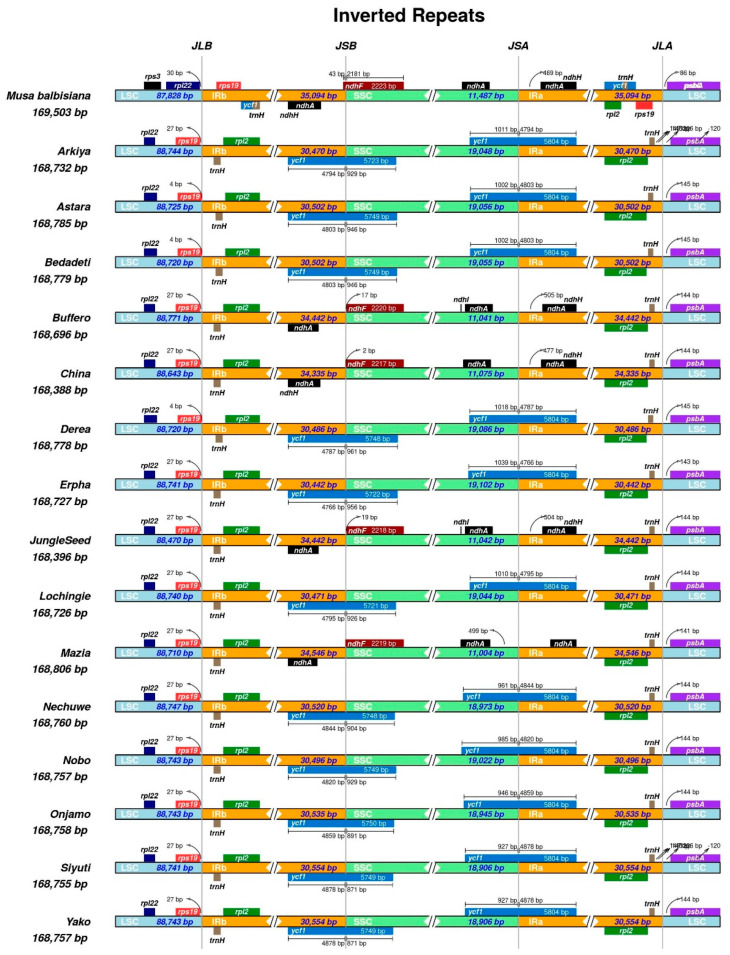
Comparative analyses of boundary’s regions (LSC, SSC, and IR) among 15 CP genomes of diverse *E. ventricosum* landraces and reference Musa CP genome.

A comparison of the border positions of the four chloroplast (CP) regions across 15 *Ensete* landraces and one *Musa* species as a reference was conducted (Figure 3). The junctions between the large single-copy (LSC), small single-copy (SSC), and inverted repeat (IR) regions (JLB, JSB, JSA, and JLA) were analyzed to identify structural variations.

Across all *E. ventricosum* landraces, the IR boundaries exhibited slight shifts, particularly at the JSB and JSA junctions. The *ycf1* and *ndhF* genes were found at the IRb/SSC (JSB) and SSC/IRa (JSA) junctions, respectively, with variations in their extensions or contractions. The reference *Musa balbisiana* genome displayed a different pattern, with the *ndhF* gene partially extending into the SSC and the *ycf1* gene showing a larger overlap within the IR region.

Among the *Ensete* landraces, the LSC/IRb (JLB) and IRa/LSC (JLA) junctions were relatively conserved, with the *rpl22* and *psbA* genes located near these boundaries. However, slight shifts in IR expansion or contraction were observed, particularly in landraces such as *Buffero* and *JungleSeed*, where the IR regions extended further into the LSC or SSC.

**Figure 4:**
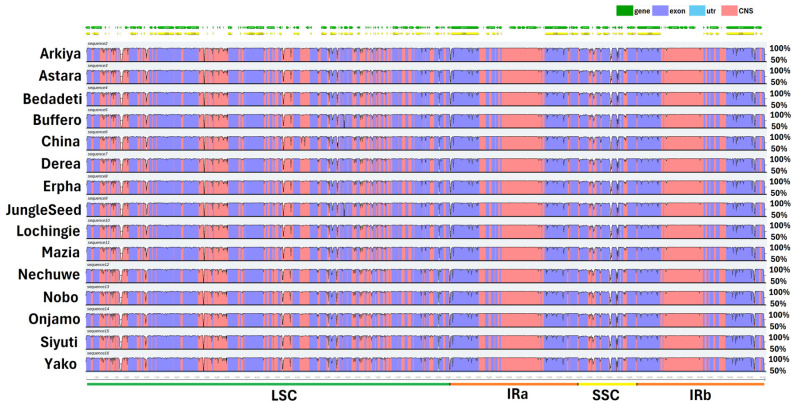
The complete chloroplast genomes of 15 *Ensete ventricosum* landraces were visualized using *Musa balbisiana* as the reference genome. Green arrows represent genes and their orientations, while yellow arrows indicate coding sequences (CDSs). Purple bars denote exons, sky-blue bars indicate untranslated regions (UTRs), and red bars represent non-coding sequences (CNSs). The vertical axis represents the percentage of sequence identity, while the genotype names are listed on the left vertical axis.

A comparative analysis of the 15 *Ensete ventricosum* landrace chloroplasts was conducted using the mVISTA tool, with *Musa balbisiana* as the reference genome. The results (Figure 4) demonstrate patterns of sequence conservation and divergence across the *E. ventricosum* chloroplast genomes. The alignment reveals a high degree of conservation in coding sequences (depicted in purple), while non-coding regions (CNS, shown in red) exhibit greater sequence variation. Notably, the inverted repeat (IR) regions (IRa and IRb) are the most conserved, displaying near-complete sequence identity across all landraces, consistent with their role in genome stabilization. In contrast, the large single-copy (LSC) and small single-copy (SSC) regions exhibit higher sequence divergence, particularly within intergenic spacers and non-coding regulatory regions. Several regions within the LSC and SSC show substantial sequence variability, as indicated by dips in the identity plot, suggesting potential hotspots for mutations, insertions/deletions (indels), or structural rearrangements. Such variations may contribute to adaptive evolution and genetic differentiation among landraces, providing valuable insights into their phylogenetic relationships and evolutionary dynamics.

Nucleotide diversity (Pi) across the *Ensete* cp genomes ranged from 0.095 to 0.0 in the coding region and 0.16 and 0.0 in intergenic regions. The five most polymorphic genes were *rrn4.5*, *psbL*, *ndhA*, *trnQ-UUG*, and *trnI-AAU*, and the most diverse intergenic regions were *ndhG-ndhl*, *psaC-ndhE*, *trnMCAU-rps14*, *rps3-rpl22*, and *rrn16-trnEUUC* (Figure S2).

### 2.2. Codon Usage and Amino Acid Frequency

Relative synonymous codon usage (RSCU) analysis was performed with the whole CP genomes and is presented in Table S3, and the results reveal that 56,067 to 56,501 codons were encoded by the entire genomes. Among these, Leucine and Serine are the most frequent amino acids, while Tryptophan is the least frequent (Figure S3). The codons with A/T at the 3′ end were more abundant than codons with G/C at the 3′ end. Codons that contain A/T at the 3′ end mostly had an RSCU value between 6 and 43, where codons with G/C had an RSCU value between 4 and 41 (Table S3). In general, the amino acid frequency and codon usage were very similar among the 15 *Ensete* landraces.

### 2.3. Transcriptional Evidence in Ensete CP Genes

**Figure 5:**
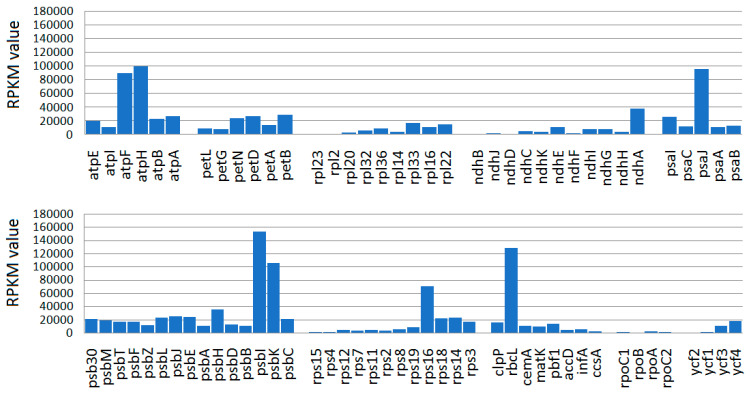
The expression patterns of the *E. ventricosum* CP genes. The expression values are normalized in RPKM (reads per kilobase per million mapped reads) on the *Y*-axis.

The expression patterns of protein-coding *E. ventricosum* chloroplast genes were studied using 41,726,332 leaf transcriptome sequences. We mostly used CDS/gene feature annotations to calculate FPKM, RPKM, and TPM values. A total of 1,058,329 reads were mapped on the *E. ventricosum* CP genome (reference Jungle seed) with an average 796 read depth. We found that the assembled consensus sequences from the mapped leaf transcriptome were 150,726 bp long, which covers 92% of the reference *Ensete* CP genomes. A large portion of reads were derived from the RuBisCo (Ribulose-1,5-Bisphosphate Carboxylase/Oxygenase) large subunit (157,249; 18%) and ATP synthase (34,978; 4%) genes. Most of the CP genes were found to be expressed in an FPKM value range of 8 to 152,959, while the *psbl*, *rbcL*, *psbK*, and *atpH* genes were expressed with an RPKM value greater than 100,000 (Figure 5).

### 2.4. Ensete Chloroplast RNA Editing

A total of 77 putative RNA editing sites were predicted in the *Ensete* cp genomes. Among these, 20 and 57 sites altered the first and second nucleotide positions of the codon, respectively. All detected changes were of the C-to-U type, resulting exclusively in nonsynonymous amino acid substitutions in protein-coding genes. Several genes, including *ndhB*, *ndhD*, *ndhF*, and *rpoB*, exhibited a relatively high number of editing sites (Table S4). These genes are involved in photosynthetic electron transport and transcriptional processes, suggesting that RNA editing may play an important role in maintaining functional integrity and regulating chloroplast activity in *Ensete*.

### 2.5. Repeat Characterization

**Figure 6:**
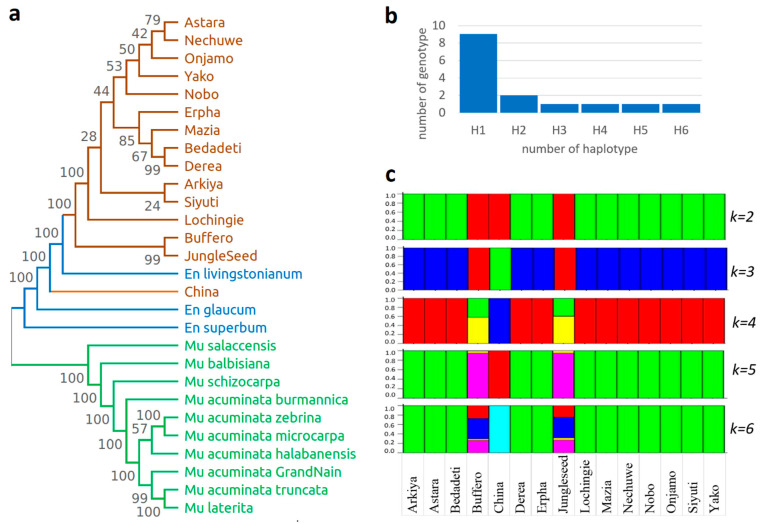
Phylogenetic relationships, haplotype distribution, and population structure of 15 Ensete landraces: (**a**) Phylogenetic tree based on chloroplast genome sequences showing relationship among 15 Ensete landraces (in brown) alongside other Ensete (blue) and Musa (green) species used as outgroups. Bootstrap values are shown at nodes. (**b**) Haplotype distribution of 15 Ensete landraces, showing six haplotypes (H1–H6). Majority of genotypes belong to H1, while remaining haplotypes are represented by only one or two genotypes each. (**c**) Population structure analysis (STRUCTURE) of 15 Ensete landraces at K = 2 to K = 6. Each vertical bar represents individual landrace, and colors indicate proportion of genetic ancestry from each cluster. Clear sub-structure is observed with increasing K values, revealing genetic differentiation among landraces.

A total of 1158 SSRs were identified across the 15 landraces of the *Ensete* chloroplast genome, with individual genomes containing 69–72 SSRs and having a density of 0.41–0.44 SSRs/kb. Six different types of SSR loci were found in all the chloroplast genomes, and the length of the SSR loci was between 10 and 34 bp. Mono- and tetranucleotide repeats were the most frequent, whereas penta- and hexanucleotide repeats were the least common (Figure 6a). On average, 71%, 7%, and 11% of SSR loci were situated in LSC, SSC, and IR regions, respectively (Table S5). The majority of mono-, di-, and trinucleotide SSRs are composed of A/T, AT/AT, and AAG/CTT motifs, respectively. Intergenic regions are richer in SSR loci than genic regions.

Tandem repeats ranged from 7 to 101 bp, with shorter repeats (7–20 bp) being the most frequent, while repeats longer than 50 bp were rare (Table S6 and Figure 6b). In total, 15,000 non-overlapping dispersed repeats were detected, including 5476 forward, 3036 reverse, and 1787 complement repeats, and the remainder were palindromic repeats (Table S7). Forward and palindromic repeats were more abundant than other types (Figure 6c).

### 2.6. Genome-Wide SNP Distribution

Chloroplast genome-wide variation among the *E. ventricosum* landraces and within the species revealed a total of 2443 variations, including 2437 SNPs and 6 indels (insertions/deletions) with a variation density of 14 per 1 kb (Table S8-1). Group-wise variations (within *Ensete* landraces and among the *Ensete* species) were visualized on the reference genome (Figure S3). As expected, the variation was low within *Ensete* landraces and among the *Ensete* species. Based on location, the greatest SNP and indel variations were found in the LSC regions. Variations were categorized by allele types (Table S8-1), and the results showed that A/G- and T/C-type variations were the most frequent. Overall, 70% of the SNP variations were found to be transversions, meaning that mutations within the same type of nucleotide were greater in the *Ensete* chloroplast genome than those from a pyrimidine to a purine or vice versa. A total of 26 protein-coding genes were found with SNP/indel variants within the landraces (Table S8-2).

### 2.7. Molecular Marker Potentiality

A total of 1078 SSRs were identified in the 15 *Ensete* chloroplast genomes, which were clustered into 72 non-redundant SSRs; they could be valuable sources for molecular markers. In order to verify their marker potential, we extracted the flanking regions of all SSR loci and designed primers: a total of 866 loci were suitable for primer design, and among them, 299 were non-redundant primers, and 59 pairs were found to be common to at least 10 *Ensete* chloroplast genomes (Table S9). Among them, 53 primer pairs were mapped onto the nuclear genome of *Ensete* and *Musa* species. Finally, 15 primer pairs were selected based on the in silico results for further wet-lab validation. All produced the expected PCR products, and 10 showed polymorphism among the tested samples.

### 2.8. Phylogenetic Relationship, Genomic Diversity, and Structure

The phylogenetic analysis, based on whole chloroplast genome sequences from 15 *Ensete* landraces alongside related *Ensete* species (*E. glaucum*, *E. lividum*, *E. superbum*) and members of the *Musa* family, revealed a well-supported clustering pattern. The *Ensete* landraces formed a monophyletic group, indicating a monophyletic, shared evolutionary origin. However, the China sample was positioned outside this main clade, suggesting significant genetic divergence. This outgroup placement may be due to geographic isolation, an earlier divergence event, or distinct evolutionary pressures shaping its chloroplast genome. Additionally, the phylogenetic placement of *Ensete* relatives and *Musa* species provided further insights into the evolutionary relationships within the Musaceae family, reinforcing the genetic distinctiveness of *Ensete* from *Musa*. The findings emphasize the evolutionary complexity within *Ensete* and highlight the need for further investigation into its biogeographic history and genetic diversity.

A chloroplast genome analysis of 15 *Ensete* landraces also revealed limited haplotype diversity (Figure 6b). Six different haplotypes (H1–H6) were detected. The most frequent one was haplotype H1, which was present in nine landraces, reflecting a very high degree of genetic similarity in most accessions. The other haplotypes (H2–H6) each corresponded to a single genotype, which reflects the presence of unique or rare variants in the collection.

Population structure analysis under a non-admixture model revealed definite genetic stratification among the 15 landraces (Figure 6c). When K = 2, the landraces grouped into two principal genetic clusters. With a further increase in K from 3 to 6, there was finer-scale differentiation revealed by the appearance of additional subgroups. The discrete clustering of landraces without admixture evidence shows that each landrace is made up mostly of a single lineage. Interestingly, landraces like Buffero, China, and Jungleseed consistently clustered together, implying that they share distinctive maternal genetic origins compared to the rest of the group. In contrast, a number of landraces like Astara, Arkya, and Yako stayed under the same cluster for all values of K, which suggests a close relationship.

## 3. Discussion

### 3.1. Chloroplast Genome Assembly, Features, Annotation, and Comparative Genomics

NGS technology can feasibly be used to produce chloroplast sequences read in parallel with the nuclear genome [29]. In this study we assembled a chloroplast genome of 15 diverse *E. ventricosum* landraces from the whole genome shotgun Illumina sequence data obtained from the SRA and subsequently performed comparative analyses. Chloroplast genomes exhibit a highly conserved number of genes similar in content with identical orientation even between the species [30]. *E. ventricosum* chloroplast genomes showed high similarity in genome length, gene number, gene order, and orientation among the sampled landraces. The overall structure of the *E. ventricosum* chloroplast genomes are identical to that of *M. acuminata* chloroplast genomes. The *E. ventricosum* chloroplast genome encoded 113–121 protein-coding genes that are comparable with other chloroplast genomes of its sister species and also other plant species [24,31,32,33,34]. Gene loss, intron loss, and gene duplication events are also reported in chloroplast genomes: our findings are also in good agreement with these findings [30,31,32]. We found that 25–31 genes are duplicated in the IR regions. Among these, the *rps19* and *ndhA* genes located in the border region of the IR and the remaining genes, four *rrna*-genes (*rrn16*, *rrn23*, *rrn4.5*, *rrn5*), *rpl2*, *rpl23*, *yfc1*, *yfc2*, six *trna*-genes (*trnM-CAU*, *trnl-UAU*, *trnv-GAC*, *trnA-UGC*, *trnl-GAU*, *trnX-A*), *rps7*, *rps12*, *ndhB* (two copies), and *ndhH*, were organized in between the *rps19* and *ndhA* genes within the IR regions of the 15 sampled chloroplast genomes. In *M. acuminata*, the *ndhA* gene was located in the border region of the IR, but this gene has an incomplete duplication: in Enset, we found this gene in the border region of the IR but with complete duplication. The *rpl2* and *rpl23* genes were duplicated in the IR regions of the *E. ventricosum* chloroplast genome, and these genes were also found in single-copy regions and LSC regions in many plant species including *Anchomanes hookeri*, *Zantedeschia aethiopica*, and *Durio zibethinus* [35]. The occurrence of duplicate copies of the *rpl2* and *rpl23* genes suggested the expansion of the IR region. We clearly demonstrate that the IR region is bigger compared to the IR regions found in the *Anchomanes hookeri*, *Zantedeschia aethiopica*, and *Durio zibethinus* chloroplast genomes [35]. The *ycf1* and *ycf2* genes duplicated in the IR regions in Enset, instead of SSC regions, a similar result to that reported in *Lemnoideae* (Araceae) in which the IR regions expanded and duplicated two genes *rps15* and *cf1* [36].

### 3.2. Codon Usage and Amino Acid Frequency

Codon usage bias provides insights into the balance between mutation pressure and natural selection in shaping genome evolution [37]. Consistent with previous studies [12,37,38], *Ensete* chloroplast genomes showed a strong preference for A/T-ending codons. This bias may be linked to the AT-rich nature of chloroplast genomes and selection for translational efficiency. Interestingly, variation in codon usage among landraces was minimal, suggesting that evolutionary constraints on chloroplast protein synthesis are conserved across Ethiopian *Ensete*. However, subtle differences in codon bias may reflect adaptive pressures, as reported in *Musa* species and other angiosperms [39]. Leucine was the most frequently encoded amino acid, which is consistent with other plant chloroplast genomes, which likely reflects both codon degeneracy and functional demand for this residue in chloroplast proteins.

### 3.3. Transcriptional Evidence in Ensete Chloroplast Genes

The transcriptional activity of the *E. ventricosum* chloroplast genes was investigated by mapping leaf transcriptome data onto the reference chloroplast genomes. The mapping results showed that nearly complete pseudo-chloroplast genomes were obtained from the transcriptome data. These findings suggest that multiple transcripts may be mapped to several non-functional genes as well as intergenic spacer (IGS) regions. Consequently, we can conclude that the identified *Ensete* chloroplast genes are transcriptionally active, including the pseudogenes, which also exhibited some level of transcriptional activity (Figure 4). Similar findings have been reported by Shi et al. [40], Amiryousefi et al. [41], and Silva et al. [42]. Using Northern blot analysis, Woodbury et al. [43] found that 90% of the pea chloroplast genome was transcribed. We also noted that genes from photosystems I and II are highly expressed compared to the other genes in Enset chloroplast genomes, consistent with observations of *Utricularia reniformis* [42].

### 3.4. Ensete Chloroplast RNA Editing

RNA editing is a post-transcriptional mechanism occurring in various cellular components, including the nucleus, cytosol, mitochondria, and plastids. Since its discovery (RNA editing), one study has shown that C-to-U editing predominates in plant organelles [41]. Our findings are consistent with this trend, as all RNA editing sites in Enset chloroplast genomes were of the C-to-U type. Importantly, the majority of edits occurred in genes such as ndhB, ndhD, ndhF, and rpoB, which are involved in the NDH complex of photosynthesis and chloroplast transcription machinery. Previous reports suggest that RNA editing in ndh genes contributes to the stability of photosynthetic performance under stress conditions [44,45]. Therefore, the relatively high frequency of edits in these genes may indicate their adaptive significance in Enset, a crop grown in diverse Ethiopian agro-ecological settings. The functional consequences of such RNA edits could include an altered protein structure, enhanced environmental plasticity, and the regulation of energy metabolism, which merit further investigation through experimental validation.

### 3.5. Repeat Characterization

Repeats are common features of plant chloroplast genomes, contributing to genome rearrangements, sequence divergence, and evolutionary adaptation [46]. Our study identified a substantial number of SSRs in *Ensete* chloroplast genomes, with distribution patterns comparable to other angiosperms [46,47,48]. We found that Ensete CP-SSR motifs are strongly biased toward AT. Similar results were also reported in many plant species including *Panax ginseng* [49], *Cucumis sativus* [49], *Sesamu mindicum* [50], and *M. acuminata* [2]. The predominance of SSRs in the LSC region likely reflects both its larger size and lower selective constraints compared with the IR and SSC regions. In general, the LSC region is larger than other regions of chloroplast genomes and therefore has a higher chance of containing more repeats compared to IR and SSC regions. In terms of functional and evolutionary implications, SSRs located in non-coding regions may contribute to genome plasticity and regulatory variation, whereas genic SSRs may influence gene expression or protein function. Similarly, the high abundance of palindromic and forward repeats suggests a potential role in intramolecular recombination and structural stability, in agreement with Henriquez et al. [30]. The predominance of short repeats (<20 bp) aligns with previous studies and reflects their supporting of evolutionary conservation and mutational dynamics. Notably, repeats and SSRs serve as valuable molecular markers for phylogenetic analysis, population genetics, and crop improvement. The large and diverse set of repeats identified in this study provides promising resources for developing chloroplast-based markers for *Ensete* germplasm characterization and breeding applications.

### 3.6. Nucleotide Diversity and Mutation Hotspots

Our analysis revealed clear heterogeneity in nucleotide diversity across different genomic regions of *Ensete*. Higher Pi values in intergenic regions compared to coding regions suggest relaxed selective constraints in non-coding DNA, a pattern consistent with other crop chloroplast genomes such as *Oryza*, *Zea*, and *Musa* [51,52]. Interestingly, several hotspot regions identified here, including *ndh* gene clusters and intergenic spacers, have also been reported as mutationally dynamic in other plants [53]. Such regions are particularly valuable for developing chloroplast-based molecular markers due to their ability to capture intraspecific variation. The presence of mutation hotspots may also reflect localized recombination, insertion–deletion dynamics, or selective pressures linked to environmental adaptation. These regions represent promising candidates for population genetics, evolutionary studies, and germplasm characterization in *Ensete*.

### 3.7. SNP Variation and Marker Potentiality

The number of variations (SNP/indels) within the whole chloroplast genome of 15 landraces is very low compared to the intraspecies level. Similar findings are also reported in *P. ginseng* [54] and rice [55]. Although *Ensete* chloroplast genomes were found to be highly conserved within the landraces (Figure 1a), 28 CP genes were found to be divergent among the species even within species such as *atpl*, *cemA*, *infA*, *ndhA*, and *ndhB*. Genes *infA*, *rpl22*, *rps19*, and *ndhE* were reported as divergent with large numbers of SNPs between different species [56]. Meanwhile, Fan et al. [56] reported nonsynonymous mutations in genes *atpF*, *atpE*, *ycf2*, and *rps15*. Our results also support these reports. The predominance of transitions over transversions is a typical feature of plant chloroplast genomes, reflecting underlying mutational mechanisms and selective constraints. The enrichment of variants in the LSC region suggests relaxed evolutionary constraints relative to the more conserved IR regions. SNPs within protein-coding genes, particularly those involved in photosynthesis and gene expression, may contribute to adaptive variation in Ensete.

From an applied perspective, the high density of SNPs and the identification of mutation hotspot regions highlight the potential of chloroplast-based SNP markers for germplasm identification, phylogenetics, and breeding. In silico analysis identified 15 SSR loci and multiple SNP-rich regions as candidate markers, of which 10 were experimentally validated as robust chloroplast SSR markers for *Ensete* breeding applications.

### 3.8. Genetic Diversity and Structure

Phylogenetic relationships, haplotype, and population structure analyses are useful for characterizing maternal genetic diversity as well as defining the evolutionary relationship of the 15 Ensete landraces studied here (Figure 6).

The phylogenetic relationships show (Figure 6a) clear divergences of the landraces into a monophyletic clade different from the clades for other *Ensete* and *Musa* species. The clustering confirms that the cultivated *Ensete* landraces had a shared origin from a domesticated gene pool. There were many sub-clusters with strong bootstrap support within the *Ensete* clade, which reflected different levels of divergence among the landraces. For instance, Buffero and Jungleseed consistently separated early from the rest of the accessions, which reflected a more distant relationship with the possibility of different evolutionary histories.

The analysis of haplotype diversity (Figure 6b) also verifies the phylogenetic results with evidence of a predominant haplotype (H1) present in most landraces (9 out of 15), which shows low haplotype diversity and hints at a bottleneck or founder effect affecting the maternal lineage of domesticated *Ensete*. The occurrence of five other distinctive haplotypes (H2–H6), each restricted to a single genotype, provides evidence that, despite their rarity, certain maternal lineages have survived and give evidence for ancient or region-conserved variants.

Population structure analysis under a non-admixture model (Figure 6c) supported phylogenetic clustering. The landraces merged into general clusters at lower values of K (K = 2 and K = 3), and higher K values (K = 4–6) reflected more population stratification with evidence of a sub-structure underpinning the landraces. Notably, the lack of admixture under this model suggests that each landrace is the product of a single, unique maternal lineage. Landraces like China, Erpha, and Buffero constituted distinctive clusters in all values of K, further supporting their genetic uniqueness and potential as reservoirs of as yet unexplored and unexploited diversity.

Together, the evidence from the phylogenetic analysis, haplotype structure, and non-admixture population structure indicate that the domesticated *Ensete* gene pool originates from a restricted maternal base as a consequence of the selective multiplication of a small number of founder lineages. The occurrence of the presence of rare haplotypes and the existence of unique population clusters, however, point to the presence of hidden genetic variation which can be vital for crop enhancement, conservation, and stress resistance, a feature that may become more important under changing conditions or planting in wider geographical ranges.

This study analyzed 15 *Ensete ventricosum* landraces, providing an initial overview of chloroplast variation, though it may underrepresent rare or region-specific types across Ethiopia’s diverse agro-ecologies. The separation of the Chinese accession highlights potential geographic structuring. Unique haplotypes and RNA editing patterns in genes such as *ndhB*, *ndhD*, and *ndhF* suggest adaptive differences linked to photosynthetic efficiency, stress tolerance, or other traits. The distribution of repeats, SNP hotspots, and patterns of nucleotide diversity may reflect selective pressures from altitude, soil, or climate. Broader sampling integrated with farmer knowledge could improve marker development, clarify domestication history, and guide breeding and conservation strategies.

## 4. Materials and Methods

### 4.1. Chloroplast Genome Reconstruction and Annotation

SRA data for 15 whole genome sequences of diverse accessions of *E. ventricosum* species were downloaded from NCBI (10 June 2022) using the SRA toolkit [57]. Then read quality was assessed in FastQC (https://www.bioinformatics.babraham.ac.uk/projects/fastqc/), and adapter and low-quality reads were removed. Complete chloroplast genomes were downloaded from NCBI (15 June 2022). Then Bowtie2 was used to map clean *E. ventricosum* SRA reads on the reference cp genome database with default parameters. Samtools was used to pick cp reads from the Bowtie2 alignment before assembly with NOVOPlasty. The *Musa balbisiana* complete chloroplast genome (NC_028439.1) was used as a reference cp genome for Ensete cp genome assembly. The GeSeq online tool was used for chloroplast gene annotations with default parameters to predict protein-coding genes and rRNA and tRNA genes. All tRNA genes were further verified by using tRNAscan-SE (Accessed on 29 March 2023 http://lowelab.ucsc.edu/tRNAscan-SE/). Then a cp genome map was drawn using the OGDRAW v.1.3.1 (Organellar genome draw) tool, following manual optimization [58]. Assembled CP genomes were deposited in the NCBI under the accession number OM925501 to OM925515.

### 4.2. Comparative Analysis of Chloroplast Genomes

The cp genome similarity of 15 diverse landraces of *E. ventricosum* was estimated by using Clustal-W alignments. Further chloroplast genomes were aligned with Mauve [59] to investigate intermolecular recombination events. To estimate major genomic variations located in LSC and SSC regions, the cp genome structures among *E. ventricosum* landraces were compared by mVISTA with a percent identity plot in Shuffle-LAGAN mode [60,61]. Furthermore, the nucleotide diversity (*Pi*) of protein-coding genes and intergenic regions was assessed by DnaSP v.6 [62]. Irscope [41] was used to analyze the genetic architecture of in the LSC/IRs and SSC/IRs border regions of Ensete cp genomes. A codon usage analysis was performed using the Bioinformatics online tool (Accessed on 30 March 2023 https://www.bioinformatics.org/sms2/codon_usage.html).

### 4.3. Transcriptome Sequencing, CP Gene Expression Analysis, and RNA Editing Site Prediction

Total RNA was extracted from the young fresh leaf tissue of greenhouse-grown 2-year-old *E. ventricosum* plants using the Monarch Total RNA Miniprep Kit (New EngladBioLabs, NEB #T2010), following the manufacturer’s protocol. DNase I was used to remove any genomic DNA contamination. RNA quality and quantity were evaluated using 1.5% Agarose gel electrophoresis and Nanodrop (Thermo Fisher Scientific, Waltham, MA, USA). High-quality RNA samples were sequenced commercially (NovoGene, Beijing, China). Raw reads were trimmed to eliminate vector sequences, and then poor-quality sequences (phred < 20) and short reads (less than 20 bp) were filtered. High-quality reads were mapped onto the assembled Ensete CP genome with bowtie2, applying default parameters. Then genome annotation was used to calculate reads per kilobase per million (RPKM), fragments per kilobase of exon per million fragments mapped (FPKM), and transcripts per million (TPM) for protein-coding genes. Ambiguously mapped reads were counted as partial matches for each CDS. Putative RNA editing sites of the protein-coding genes were predicted with an in silico approach using the PREP-cp database [63].

### 4.4. Repeat Structure Analysis

Repeat structure analysis was performed by using three different tools: REPuter [64], MISA v1.0 [65], and Phobosv.3.3.12 [66]. The locations and sizes of forward, reverse, palindromic, and complementary repeats were estimated by REPuter with a minimal size of 30 bp, hamming distance of 3, and over 70% identity. Simple sequence repeats (SSRs) were identified with MISA following the parameters, where the motifs consisted of one to six nucleotides, and the minimum repeat unit was defined as ten for mononucleotides, five for dinucleotides, four for trinucleotides, and three for tetra-, penta-, and hexanucleotides. Tandem repeats (7–100 bp) were identified by Phobos using default parameters.

### 4.5. SNP Calling

The CP reads of the samples were mapped onto the reference CP genome using bowtie2; then samtools was used to convert and sort SAM (Sequence Alignment/Map) into BAM (binary SAM); after that, bcftools was used for final realignment and variant identification. The raw SNP call of the variant was subsequently filtered, formatted, and summarized using bcftools and perl scripts. The SNP locus was manually verified using alignments. The SNP distribution and frequency were determined using xls and R-script. CP genome-wide SNP distribution was visualized by CIRCOS.

### 4.6. Development of Potential Molecular Markers

SSR loci were extracted with 100 bp up- and downstream for SSR primer modeling and subsequent characterization. CP-SSR primers were first mapped onto 15 Ensete CP genomes. The CP-SSR primers found to be common in at least 10 Ensete CP genomes were further mapped onto the nuclear genome of *E. ventricosum* (cv. Bedadeti, Dera, Onjamo, JungleSeed) and Musa species (*Musa acuminata*, *M. balbisiana*, *M. schizocarpa and M. itinerans*) using e-PCR. Then the results were manually inspected, and the 15 most common primer pairs were picked for wet-lab validations.

### 4.7. Phylogenic Relationship Haplotype Analysis and Structure Analysis

The chloroplast (cp) genomes of three *Ensete* species (*E. livingstonianum*, *E. glaucum*, and *E. superbum*) and ten *Musa* species were retrieved from the NCBI database. Additionally, the complete cp genomes of 15 landraces of *Ensete ventricosum* were de novo assembled. A total of 28 cp genomes (3 *Ensete* + 10 *Musa* + 15 *E. ventricosum* landraces) were aligned using the MAFFT tool with default parameters. Phylogenetic relationships were inferred using the Neighbor-Joining (NJ) method based on the Tamura–Nei genetic distance model. The reliability of the phylogenetic tree was assessed using a bootstrap analysis with 1000 replicates.

The alignments of the 15 cp genomes were imported into DnaSP6 software (version 6.12.03) for the analysis of the number of haplotypes grouped within these 15 *Ensete* landraces based on the cp genome. Subsequently, SNPs were called, exported in Nexus format, and uploaded to the Galaxy server. The SNP data was then converted to VCF format. The VCF-format SNP data was analyzed using STRUCTURE software v2.34 with non-admixture haploid genome parameters for population structure analysis, applying k values from 2 to 6 and three replications with 1000 burn-ins.

## 5. Conclusions

In this study, we assembled and analyzed the complete chloroplast genomes of 15 *Ensete* landraces from diverse Ethiopian regions, integrating structural, genetic, and transcriptomic data. The genomes showed a conserved structure and gene order, while SSRs, SNPs, and codon usage patterns revealed both common and rare variations. Highly expressed genes displayed distinct evolutionary signatures, consistent with the observed nucleotide diversity and hotspot regions. Phylogenetic and population analyses indicate a narrow maternal genetic base, yet rare haplotypes highlight hidden diversity. These integrated insights provide a robust foundation for marker development, conservation strategies, and breeding programs to sustainably utilize *Ensete*’s genetic resources.

## Figures and Tables

**Table 1:** The characteristics of the assembled chloroplast genomes of *Ensete ventricosum*.

Landraces	Genome Size (bp)	LSC Size (bp)	SSC Size (bp)	IR Size (bp)	Total No of Genes	No of Unique Genes	No of Genes Found in Duplicate Copy	tRNA (t/u)	rRNA (t/u)	Protein-Coding Gene (t/u)	Exon	Intron	GC%
Arkiya	168,732	88,743	11,076	34,414/34,495	137	105	23	37/28	8/4	91/76	56	37	37
Astara	168,785	88,724	11,080	34,461/34,516	137	105	23	37/28	8/4	91/76	56	37	37
Bedadeti	168,779	88,719	11,075	34,463/34,518	137	105	23	37/28	8/4	91/76	56	37	37
Buffero	168,696	88,768	11,040	34,442/34,254	138	105	25	37/28	8/4	92/75	56	37	37
China	168,388	88,642	11,074	34,334/34,334	135	105	22	37/28	8/4	89/75	56	37	37
Derea	168,778	88,719	11,075	34,462/34,518	137	105	23	37/28	8/4	91/76	56	37	37
Erpha	168,727	88,740	11,075	34,413/34,495	137	105	23	37/28	8/4	91/76	56	37	37
JungleSeed	168,396	88,469	11,040	34,441/34,442	138	105	24	37/28	8/4	92/76	56	37	37
Lochingie	168,726	88,739	11,080	34,410/34,493	137	105	23	37/28	8/4	91/76	56	37	37
Mazia	168,806	88,707	11,076	34,523/34,310	137	105	24	37/28	8/4	91/75	56	37	37
Nechuwe	168,760	88,746	11,076	34,439/34,495	137	105	23	37/28	8/4	91/76	56	37	37
Nobo	168,757	88,740	11,076	34,441/34,261	137	105	23	37/28	8/4	91/76	56	37	37
Onjamo	168,758	88,740	11,080	34,440/34,264	137	105	23	37/28	8/4	91/76	56	37	37
Siyuti	168,755	88,740	11,076	34,440/34,495	137	105	23	37/28	8/4	91/76	56	37	37
Yako	168,757	88,740	11,080	34,439/34,263	137	105	23	37/28	8/4	91/76	56	37	37
Musa	169,503	87,828	11,487	35,094	148	104	30	38/26	8/4	106/76	58	37	37
Average	168,707	88,712	11,098	34,437/34,410	137	105	23	37/28	8/4	91/76	56	37	37
Min	168,388	88,469	11,040	34,334/34,254	135	105	22	37/28	8/4	89/75	56	37	37
Max	168,806	88,768	11,080	34,523/34,518	138	105	25	37/28	8/4	92/76	56	37	37

Note: u represents the number of unique genes, and t represents the total number of genes.

## Data Availability

The original contributions presented in this study are included in the Supplementary Materials. Further inquiries can be directed to the corresponding authors.

## Supplementary Materials

The following supporting information can be downloaded at: https://www.mdpi.com/article/10.3390/ijms26199561/s1.
