# Supplementary material for: Chloroplast Genome Diversity and Marker Potentials of Diverse Ensete ventricosum Accessions
Source: Int J Mol Sci. 2025 Sep 30;26(19):9561. doi: 10.3390/ijms26199561 (PMC12525447; doi:10.3390/ijms26199561)
Supplement: Supplementary file 1 [file ijms-26-09561-s001.zip › 2025En15lLCP-Supplydata_Figures-v01.pdf]

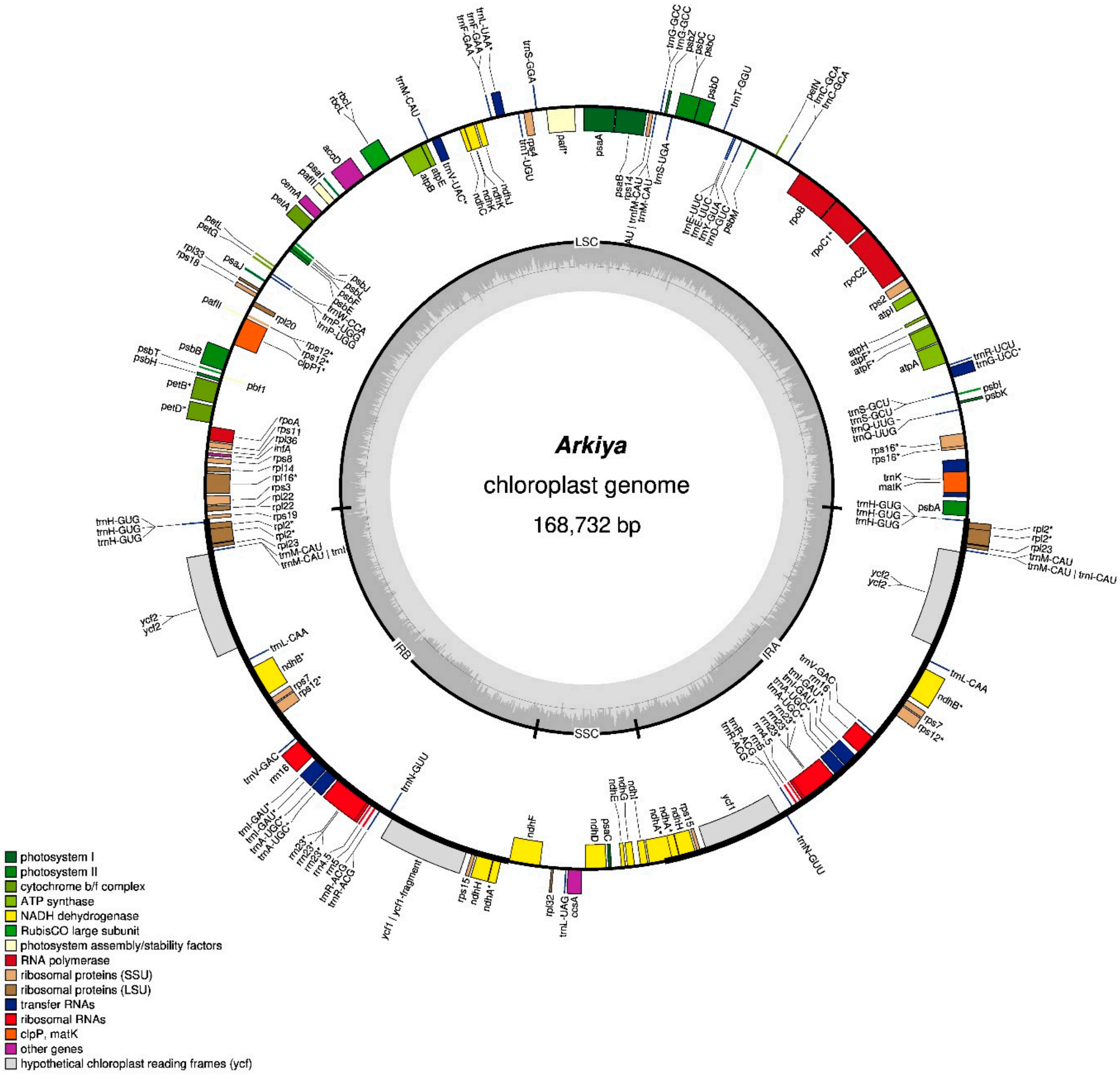

Figure S1(a) Circular map of the *Ensete ventricosum* "Arkiya"chloroplast genome. Genes with the boxes inside and outside the circle are transcribed in clockwise direction and counterclockwise direction, respectively. The inner circle indicates the GC content and inverted repeat boundaries.

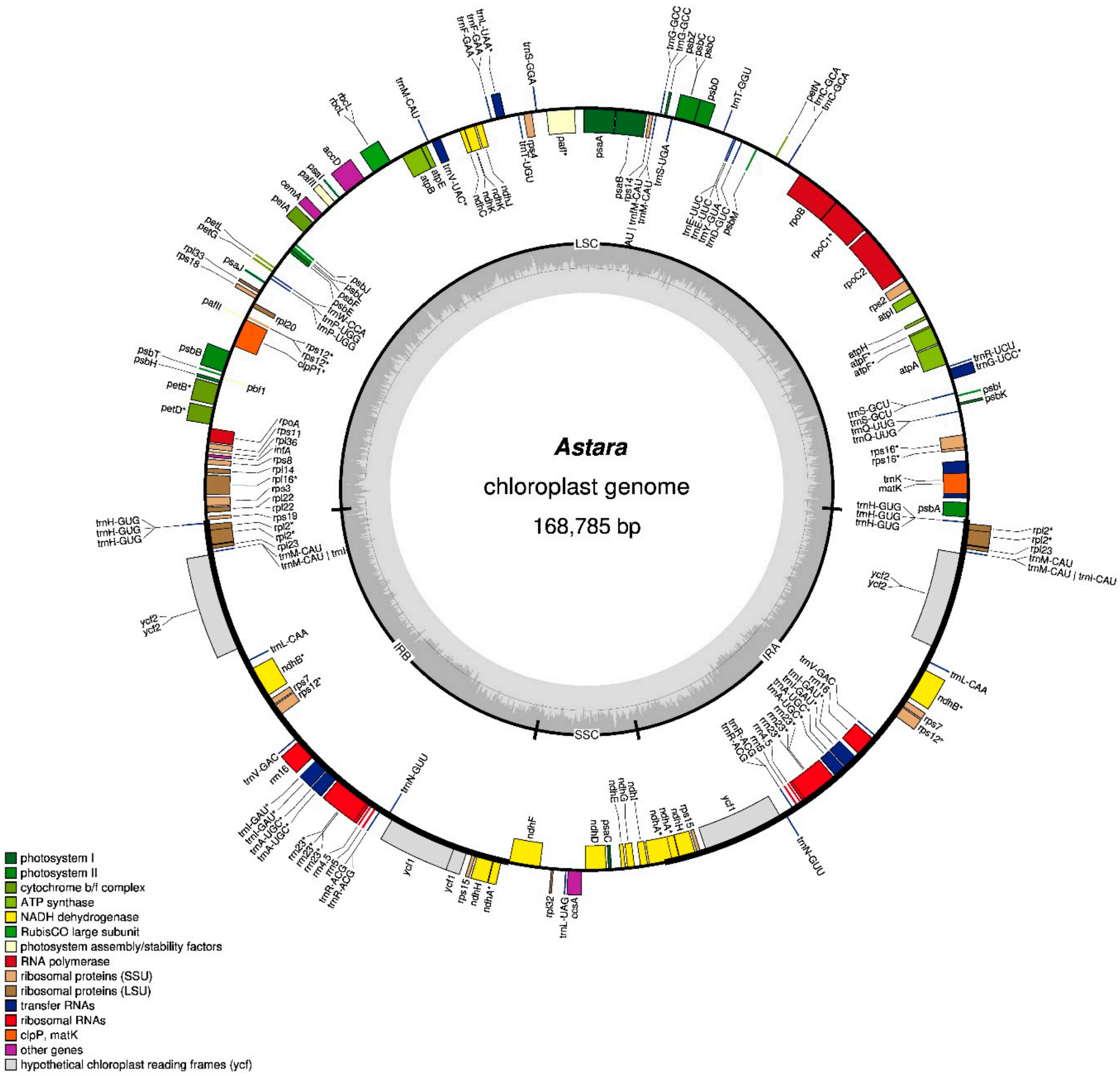

Figure S1 (b) Circular map of the *Ensete ventricosum* "Astara"chloroplast genome. Genes with the boxes inside and outside the circle are transcribed in clockwise direction and counterclockwise direction, respectively. The inner circle indicates the GC content and inverted repeat boundaries.

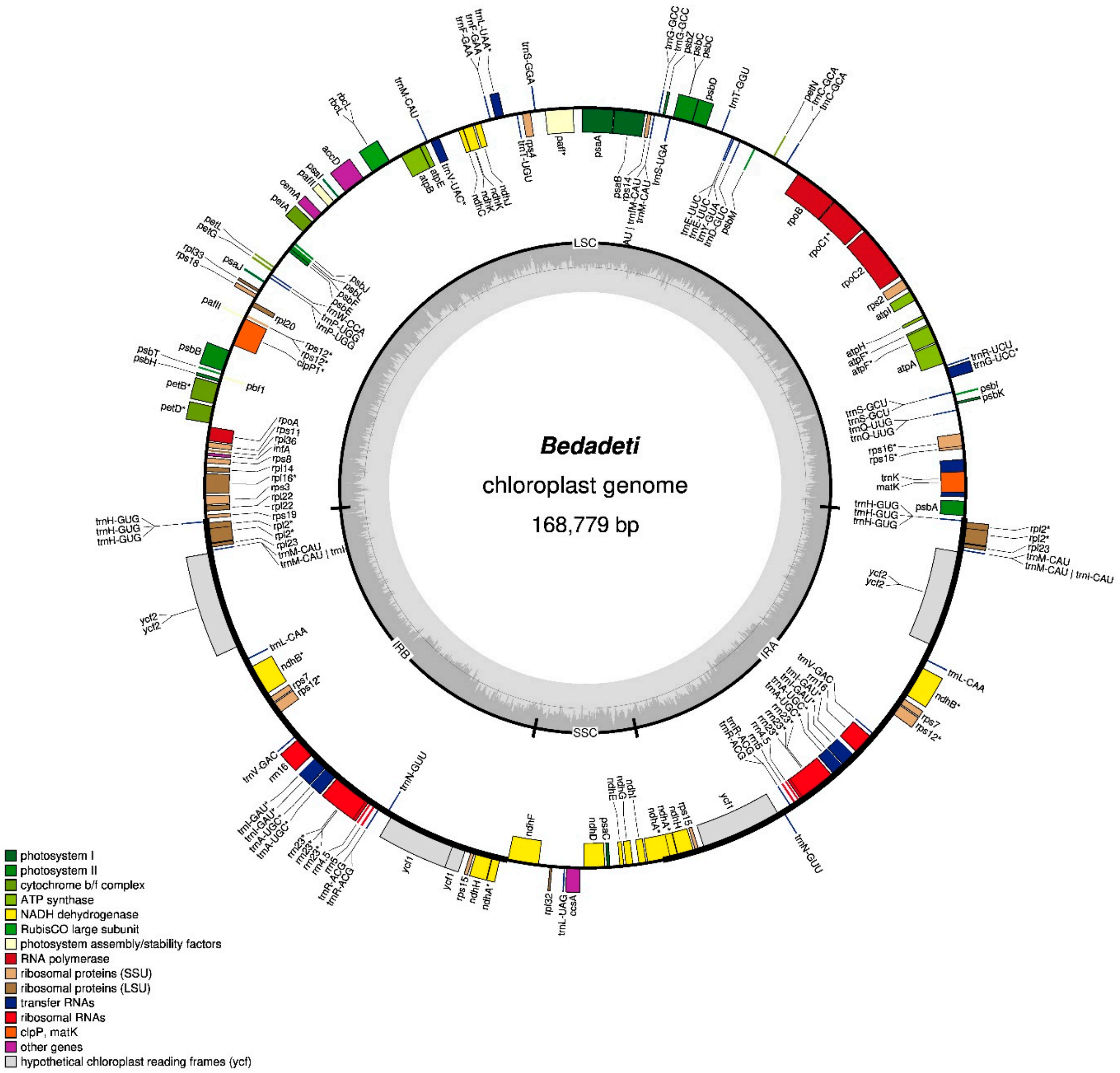

Figure S1 (c) Circular map of the *Ensete ventricosum* " Bedadeti" chloroplast genome. Genes with the boxes inside and outside the circle are transcribed in clockwise direction and counterclockwise direction, respectively. The inner circle indicates the GC content and inverted repeat boundaries.

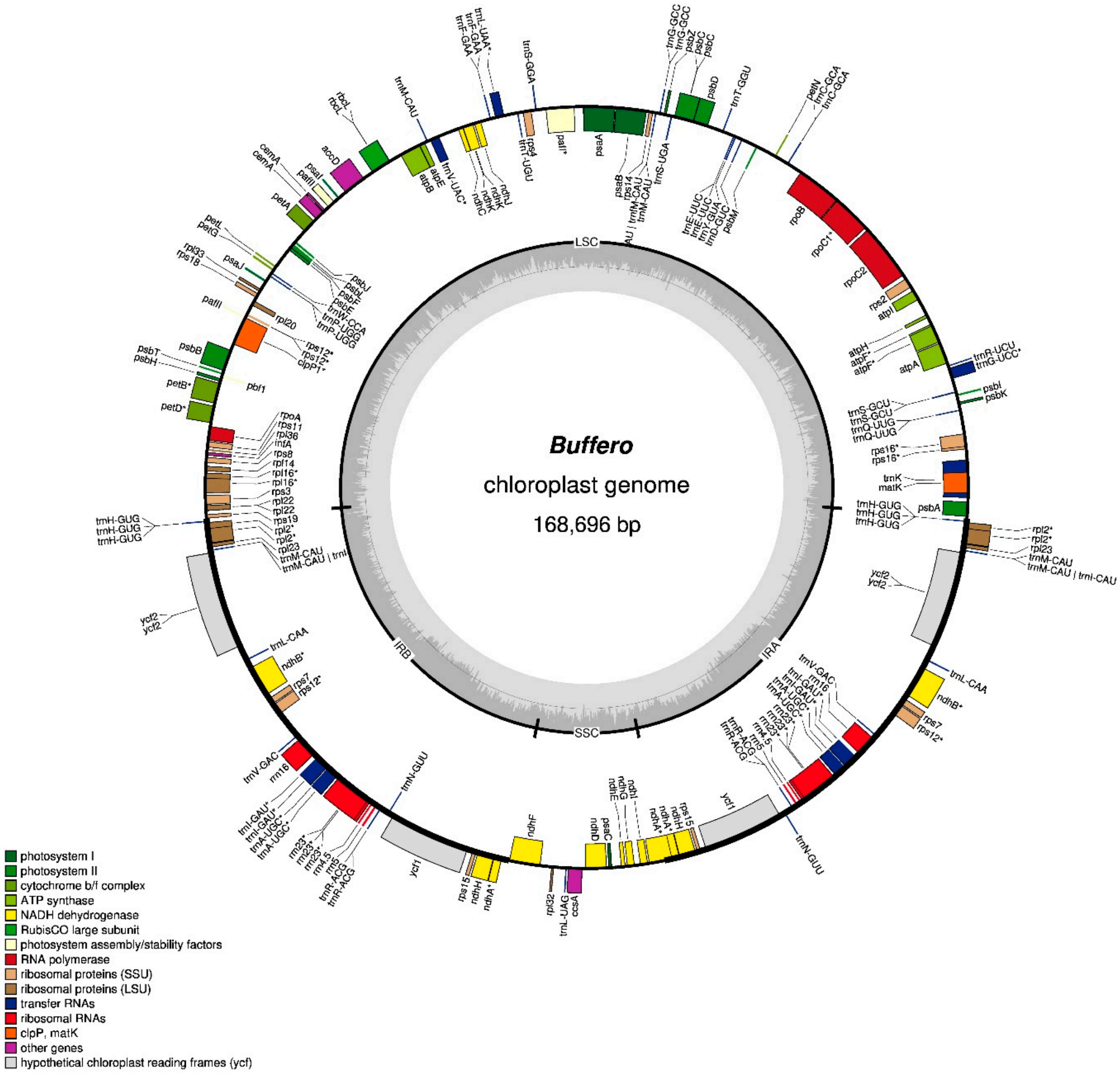

Figure S1 (d) Circular map of the *Ensete ventricosum* " Buffero "chloroplast genome. Genes with the boxes inside and outside the circle are transcribed in clockwise direction and counterclockwise direction, respectively. The inner circle indicates the GC content and inverted repeat boundaries.

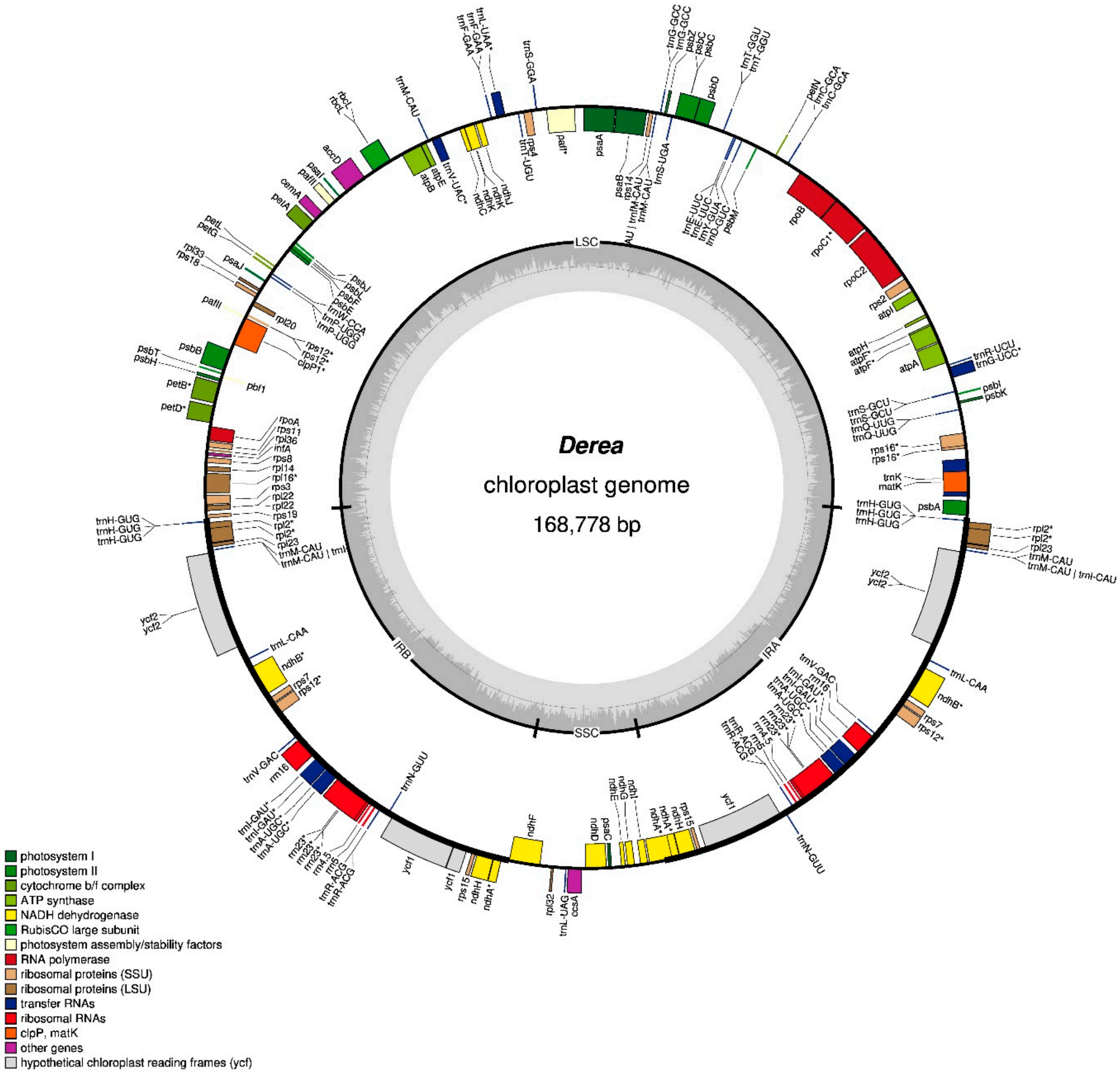

Figure S1 (e) Circular map of the *Ensete ventricosum* "Derea "chloroplast genome. Genes with the boxes inside and outside the circle are transcribed in clockwise direction and counterclockwise direction, respectively. The inner circle indicates the GC content and inverted repeat boundaries.

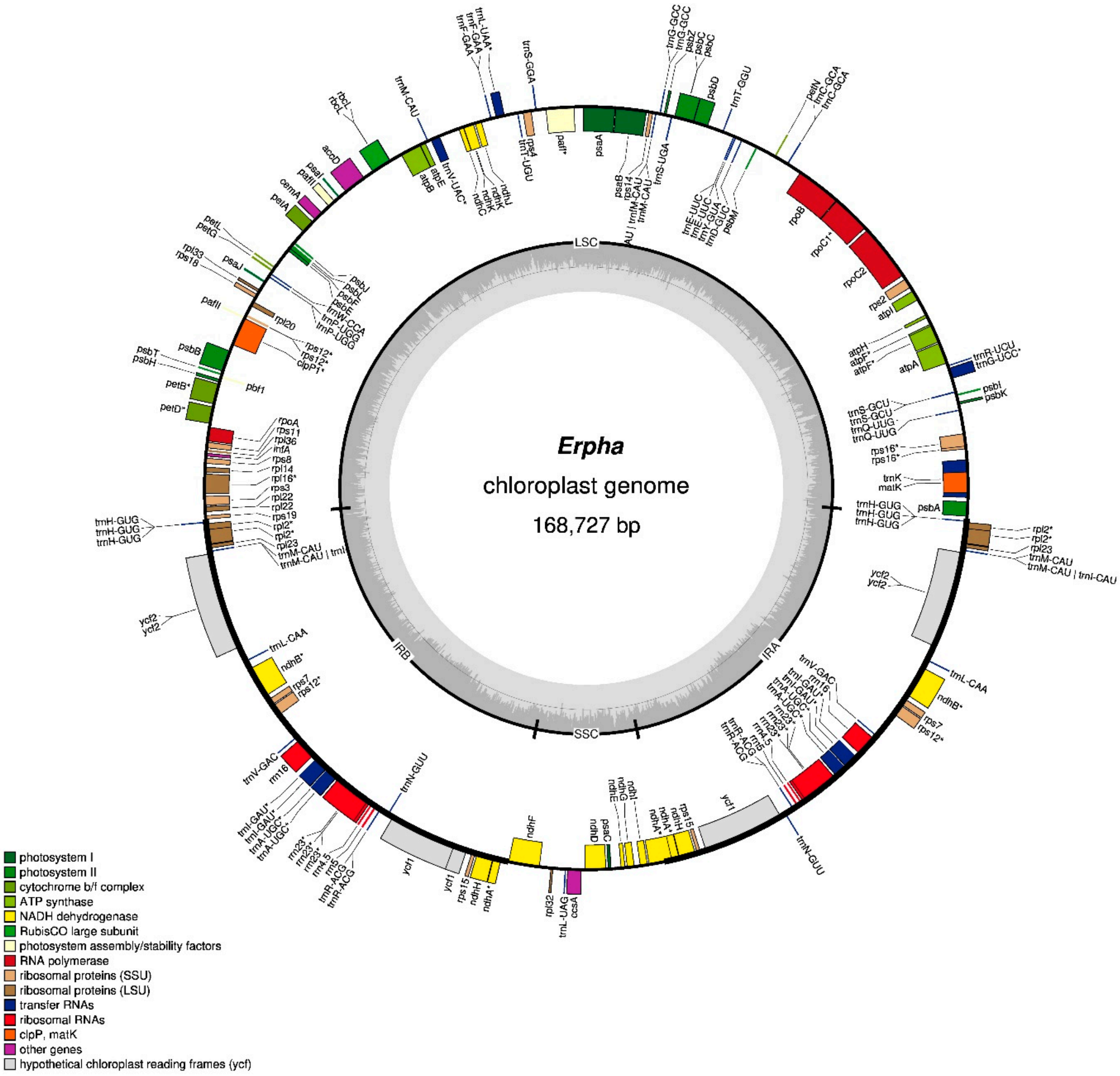

Figure S1 (f) Circular map of the *Ensete ventricosum* " Erpha" chloroplast genome. Genes with the boxes inside and outside the circle are transcribed in clockwise direction and counterclockwise direction, respectively. The inner circle indicates the GC content and inverted repeat boundaries.

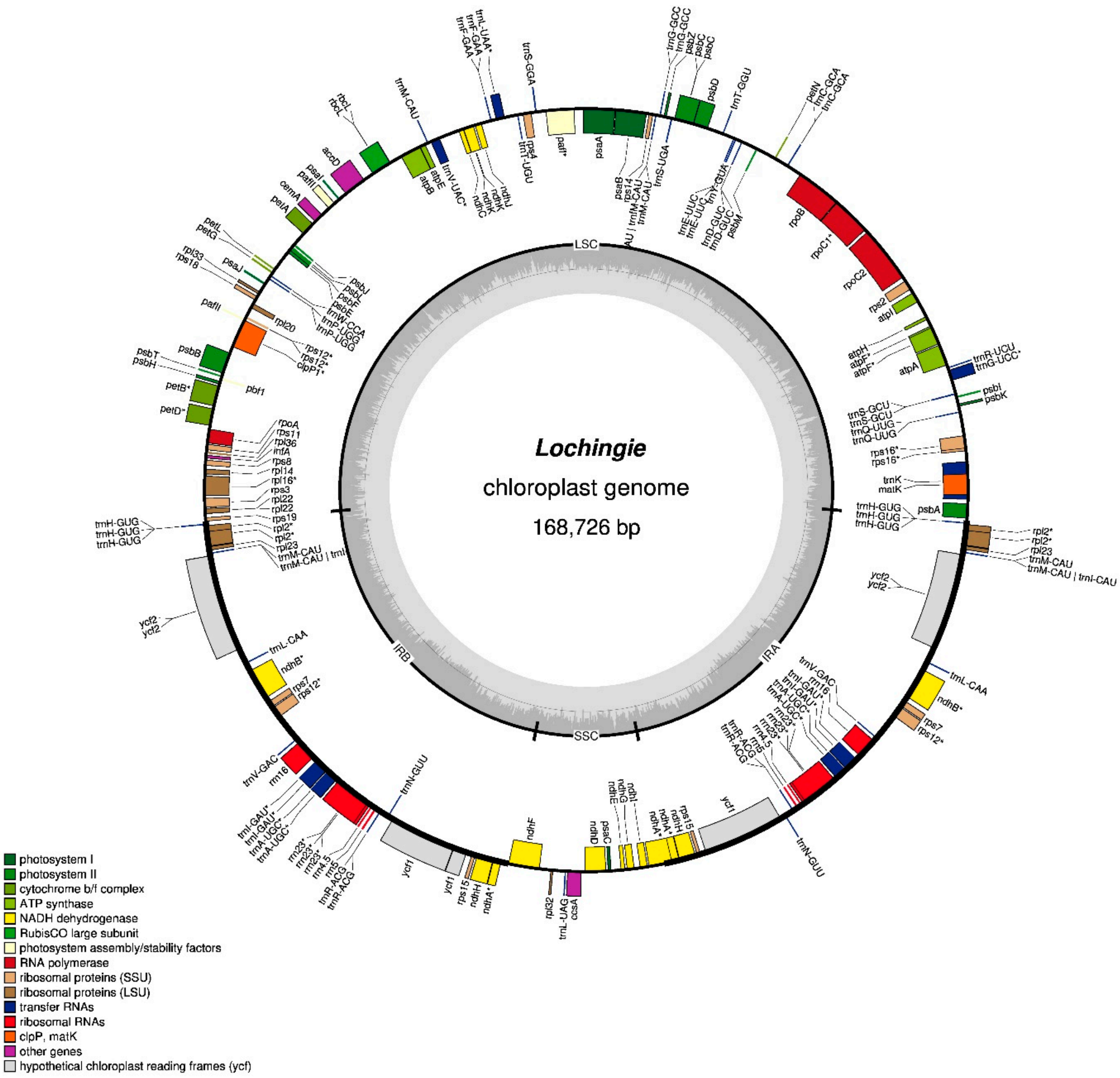

Figure S1 (g) Circular map of the *Ensete ventricosum* " Lochingie" chloroplast genome. Genes with the boxes inside and outside the circle are transcribed in clockwise direction and counterclockwise direction, respectively. The inner circle indicates the GC content and inverted repeat boundaries.

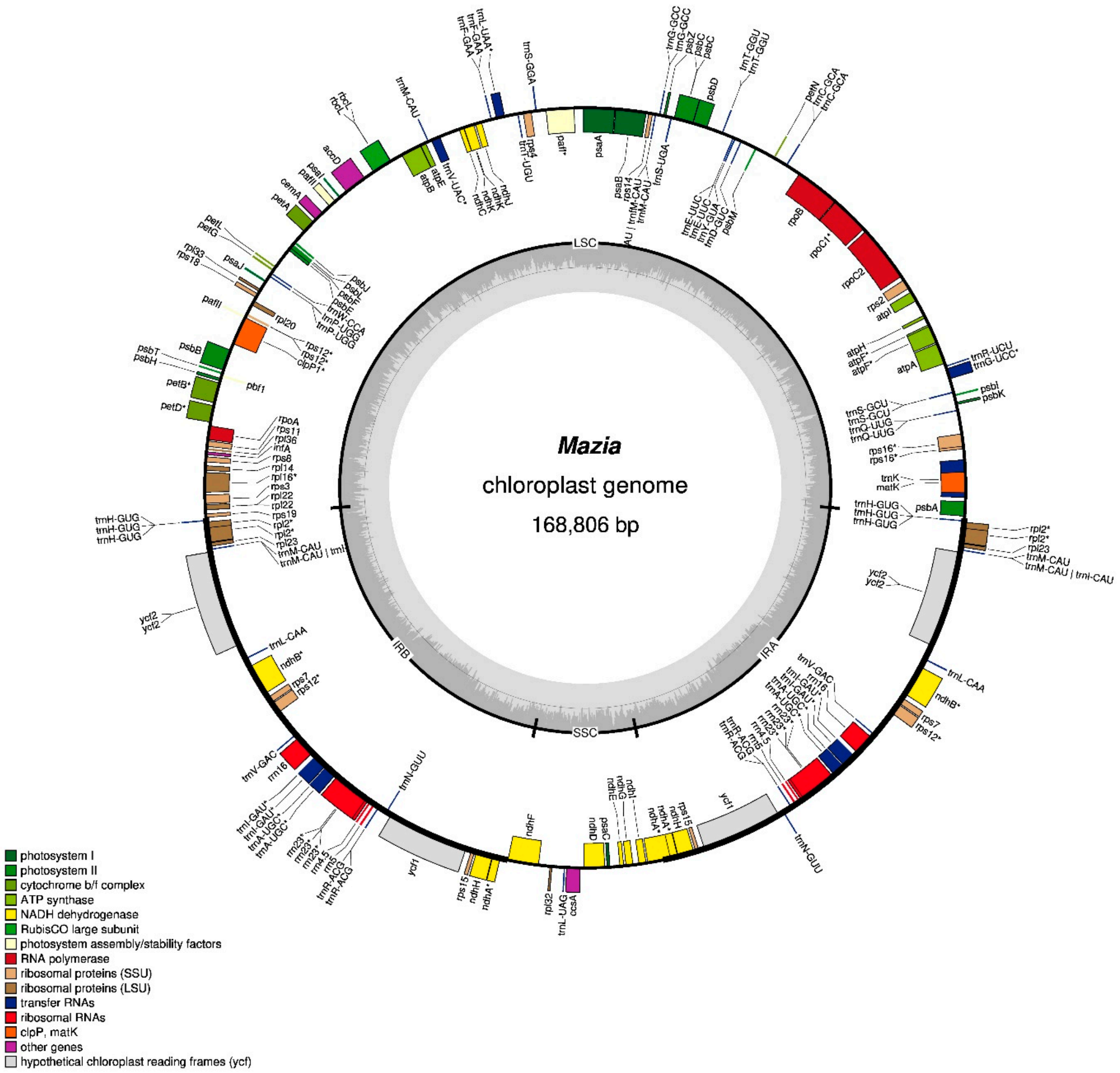

Figure S1 (h) Circular map of the *Ensete ventricosum* " Mazia" chloroplast genome. Genes with the boxes inside and outside the circle are transcribed in clockwise direction and counterclockwise direction, respectively. The inner circle indicates the GC content and inverted repeat boundaries.

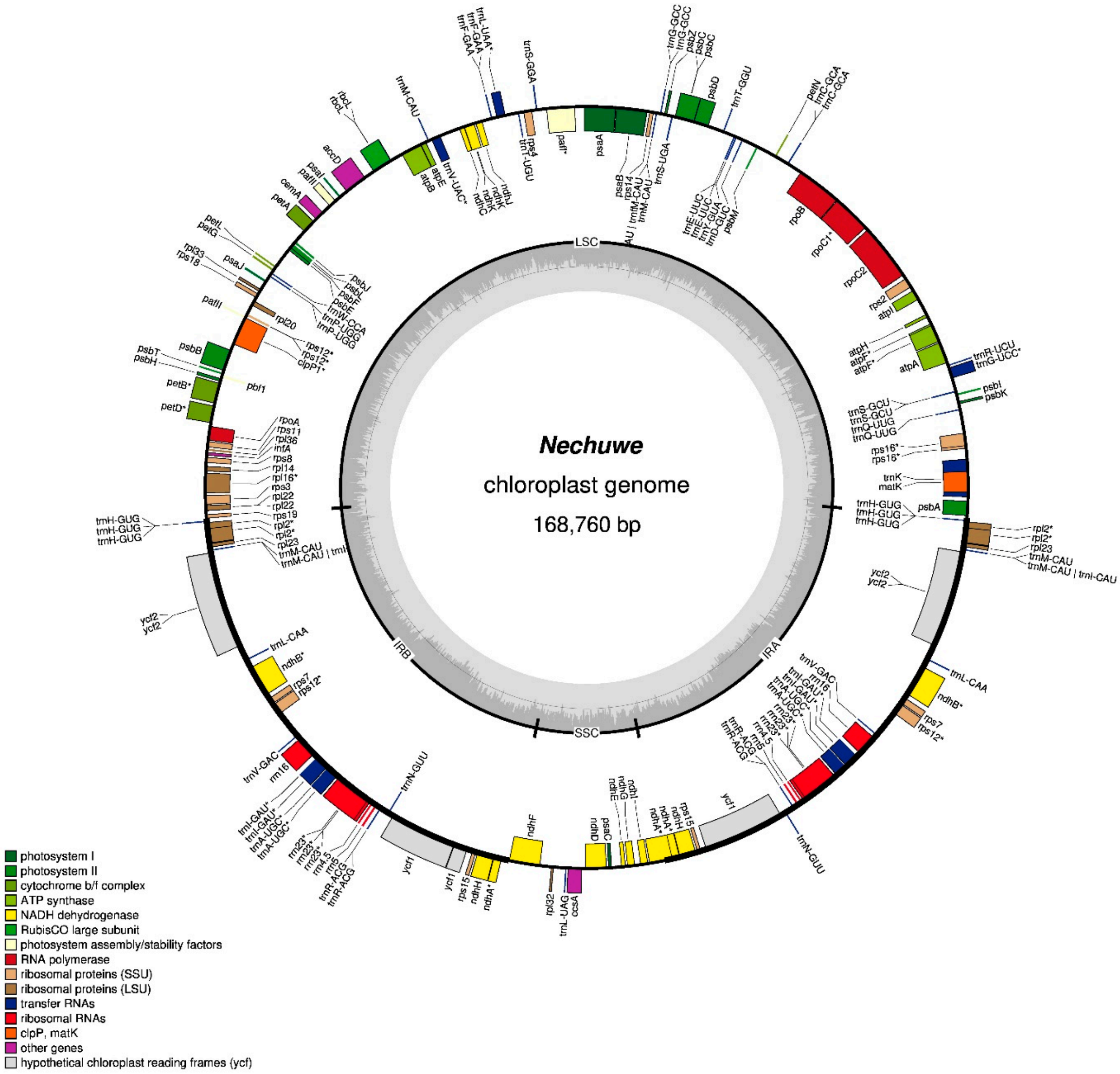

Figure S1 (i) Circular map of the *Ensete ventricosum* "Nechuwe "chloroplast genome. Genes with the boxes inside and outside the circle are transcribed in clockwise direction and counterclockwise direction, respectively. The inner circle indicates the GC content and inverted repeat boundaries.

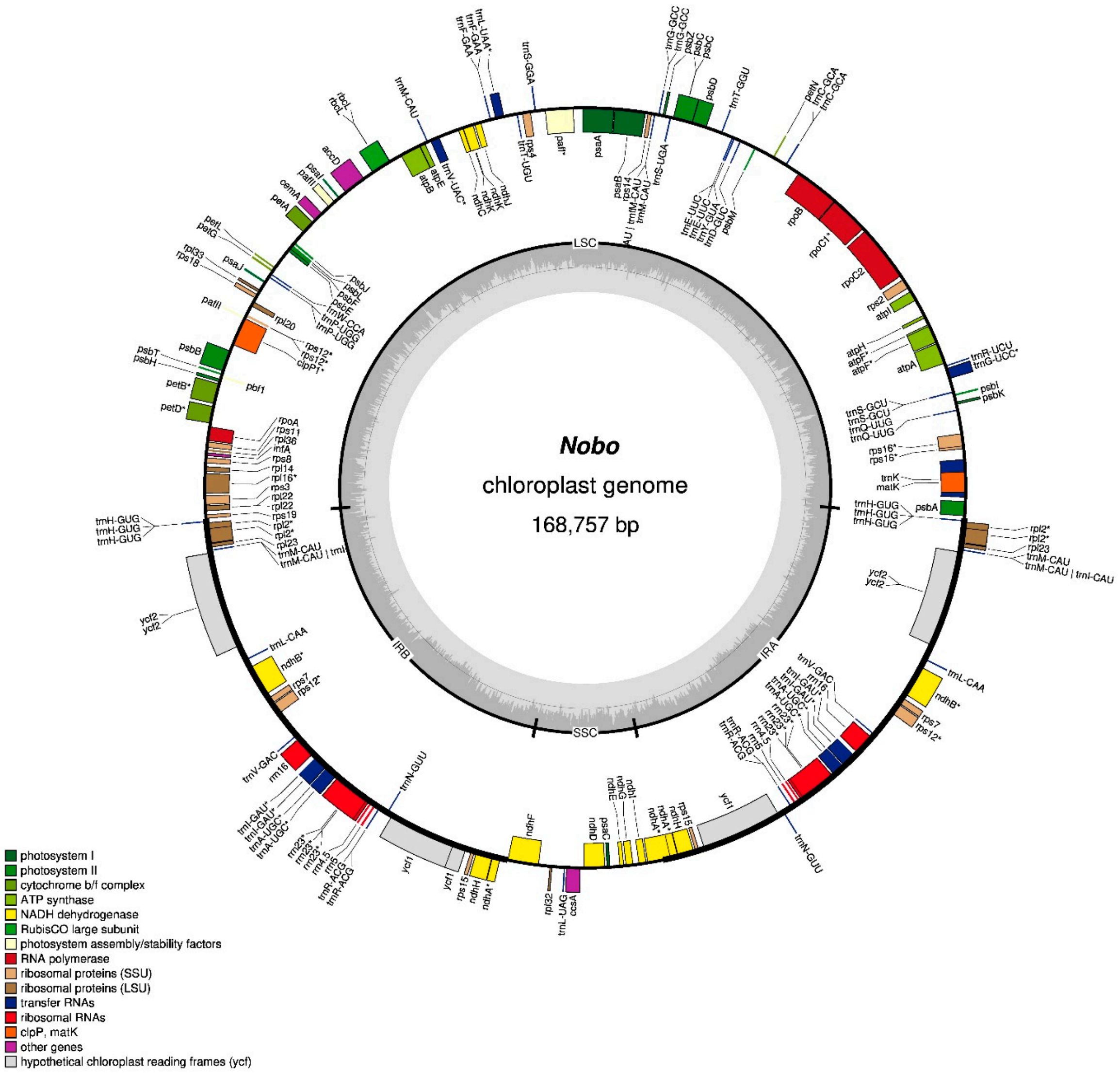

Figure S1 (j) Circular map of the *Ensete ventricosum* " Nobo"chloroplast genome. Genes with the boxes inside and outside the circle are transcribed in clockwise direction and counterclockwise direction, respectively. The inner circle indicates the GC content and inverted repeat boundaries.

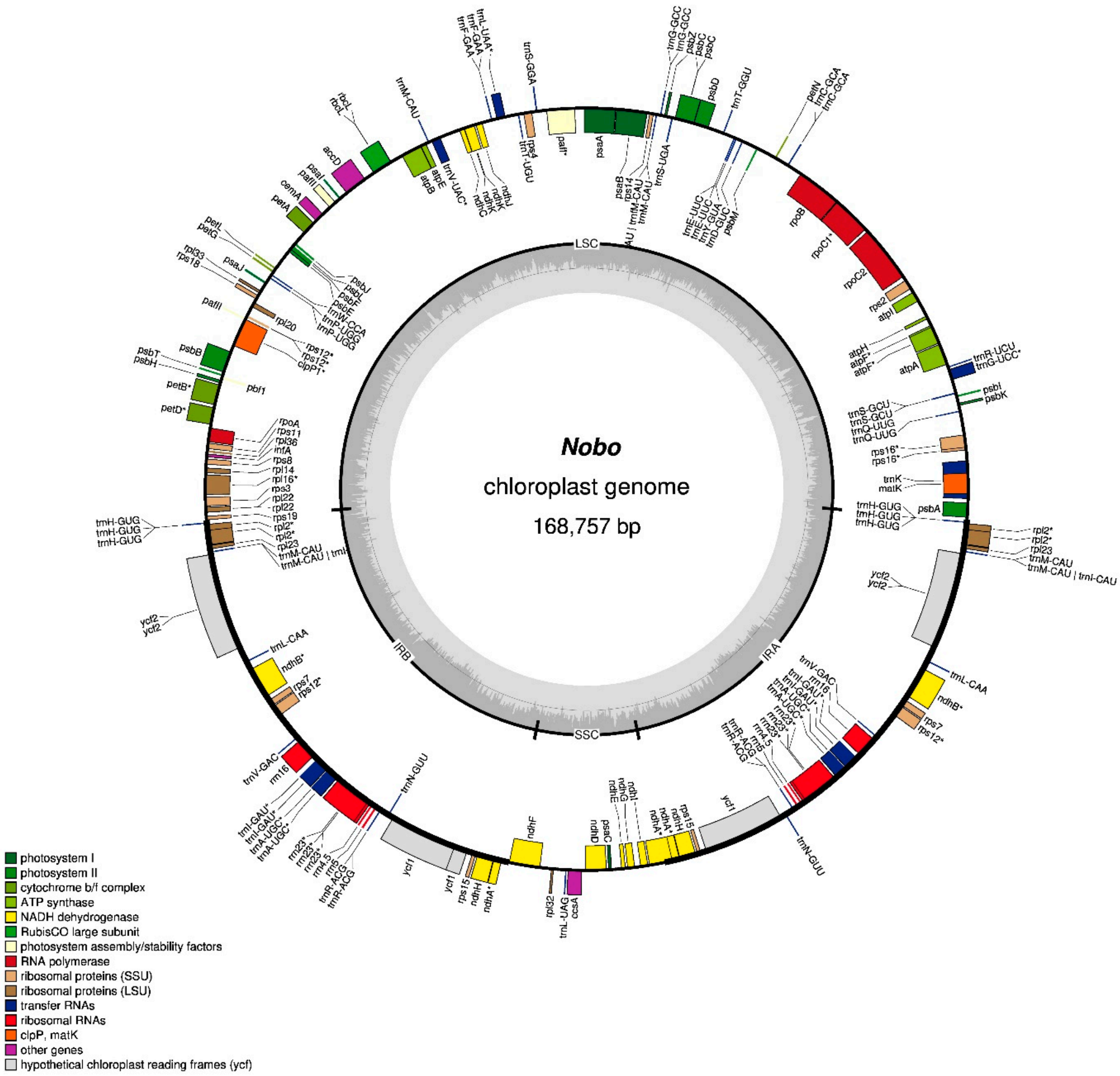

Figure S1 (k) Circular map of the *Ensete ventricosum* "Onjamo "chloroplast genome. Genes with the boxes inside and outside the circle are transcribed in clockwise direction and counterclockwise direction, respectively. The inner circle indicates the GC content and inverted repeat boundaries.

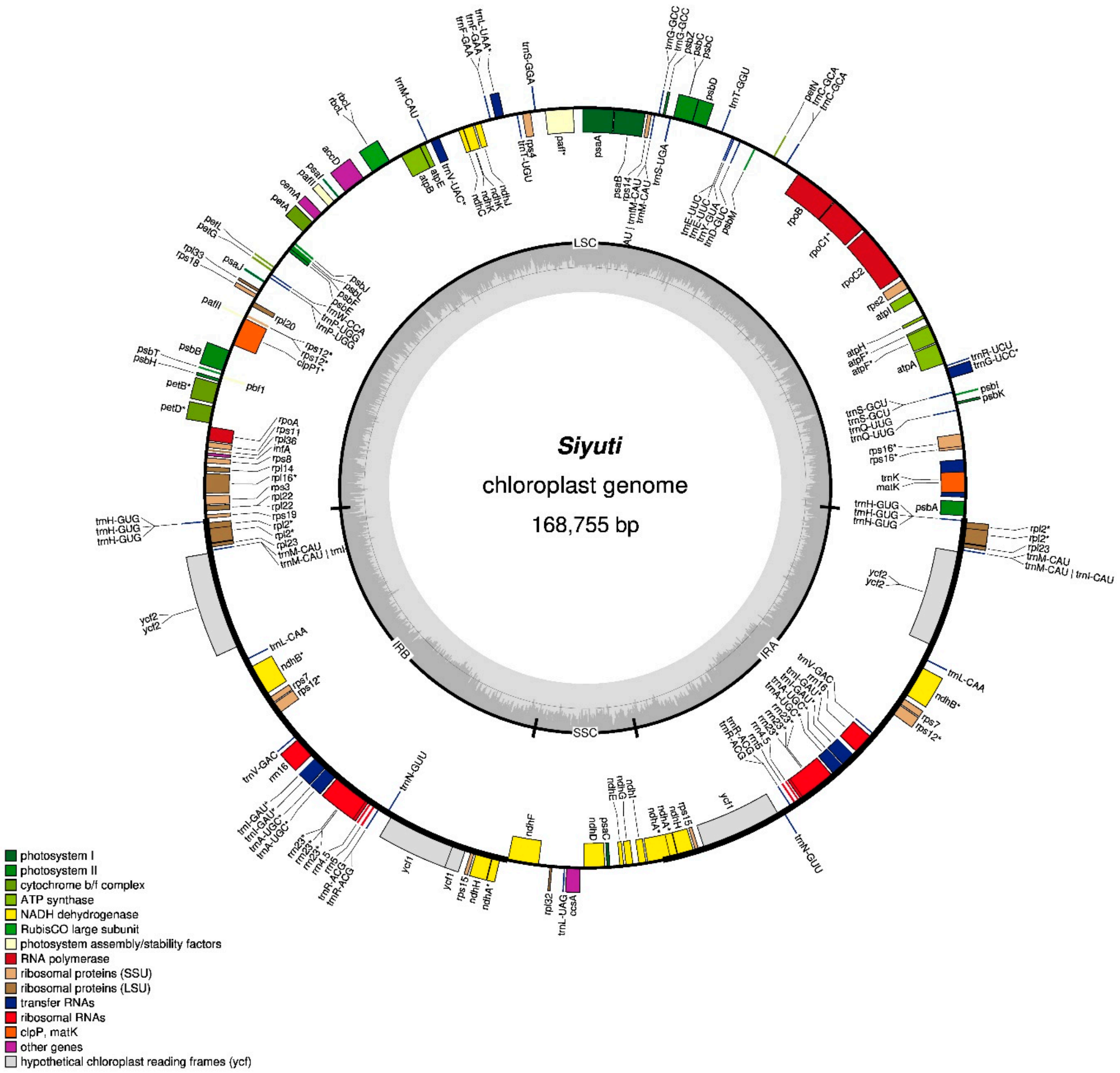

Figure S1 (l) Circular map of the *Ensete ventricosum* " Siyuti"chloroplast genome. Genes with the boxes inside and outside the circle are transcribed in clockwise direction and counterclockwise direction, respectively. The inner circle indicates the GC content and inverted repeat boundaries.

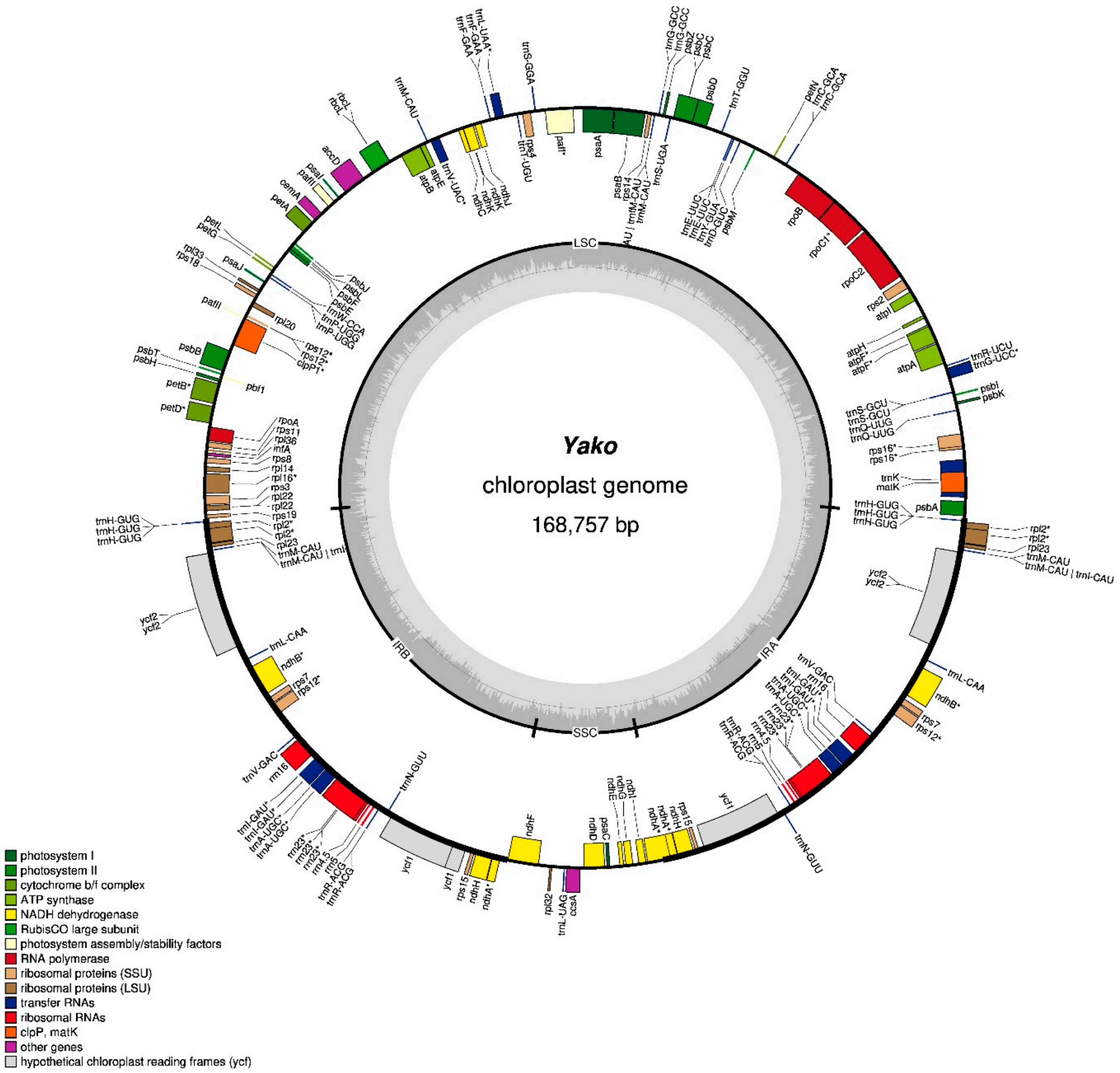

Figure S1 (m) Circular map of the *Ensete ventricosum* " Yako"chloroplast genome. Genes with the boxes inside and outside the circle are transcribed in clockwise direction and counterclockwise direction, respectively. The inner circle indicates the GC content and inverted repeat boundaries.

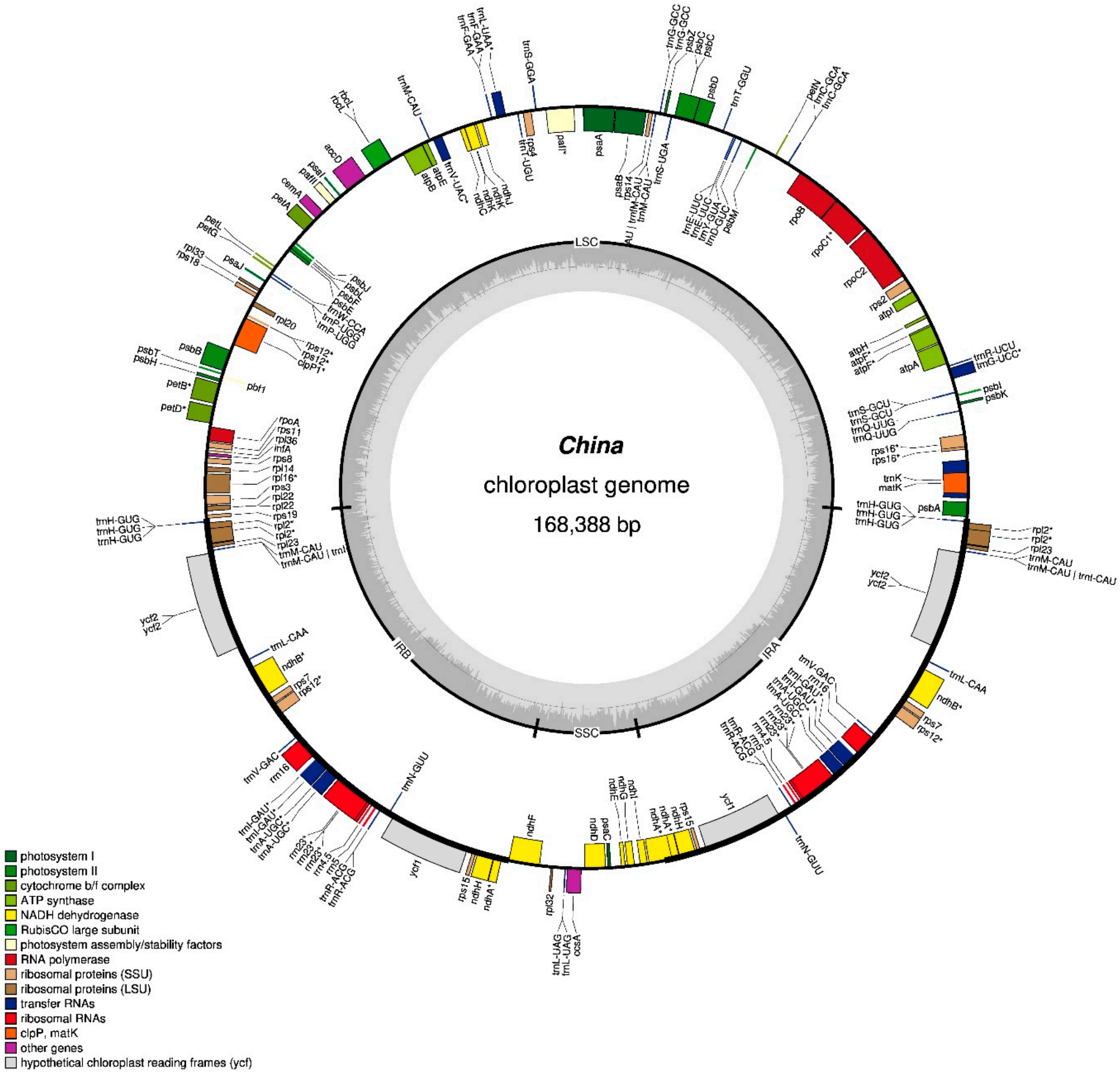

Figure S1 (n) Circular map of the *Ensete ventricosum* " China"chloroplast genome. Genes with the boxes inside and outside the circle are transcribed in clockwise direction and counterclockwise direction, respectively. The inner circle indicates the GC content and inverted repeat boundaries.

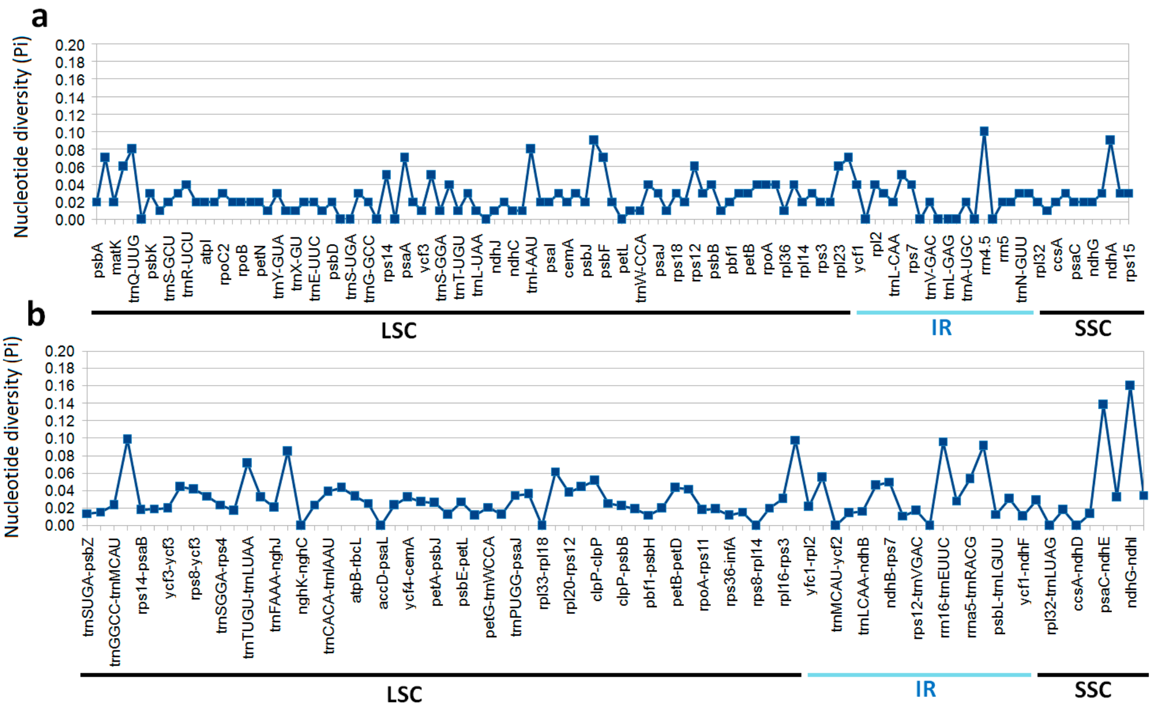

Figure S3. Nucleotide diversity (pi) value genic (a) and intergenic(b) regions of the *Ensete ventricosum* CP genome.

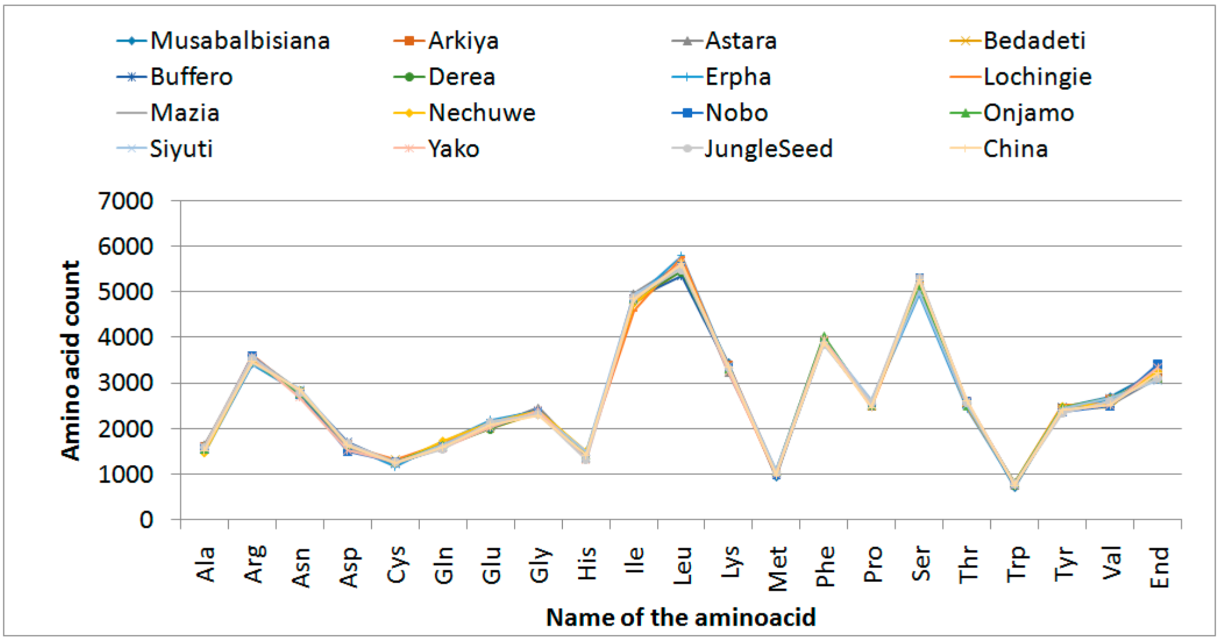

Figure S4 A comparative distribution of 20 amino acids and stop codons in the protein-coding genes of 15 diverse *Ensete ventricosum* landraces.

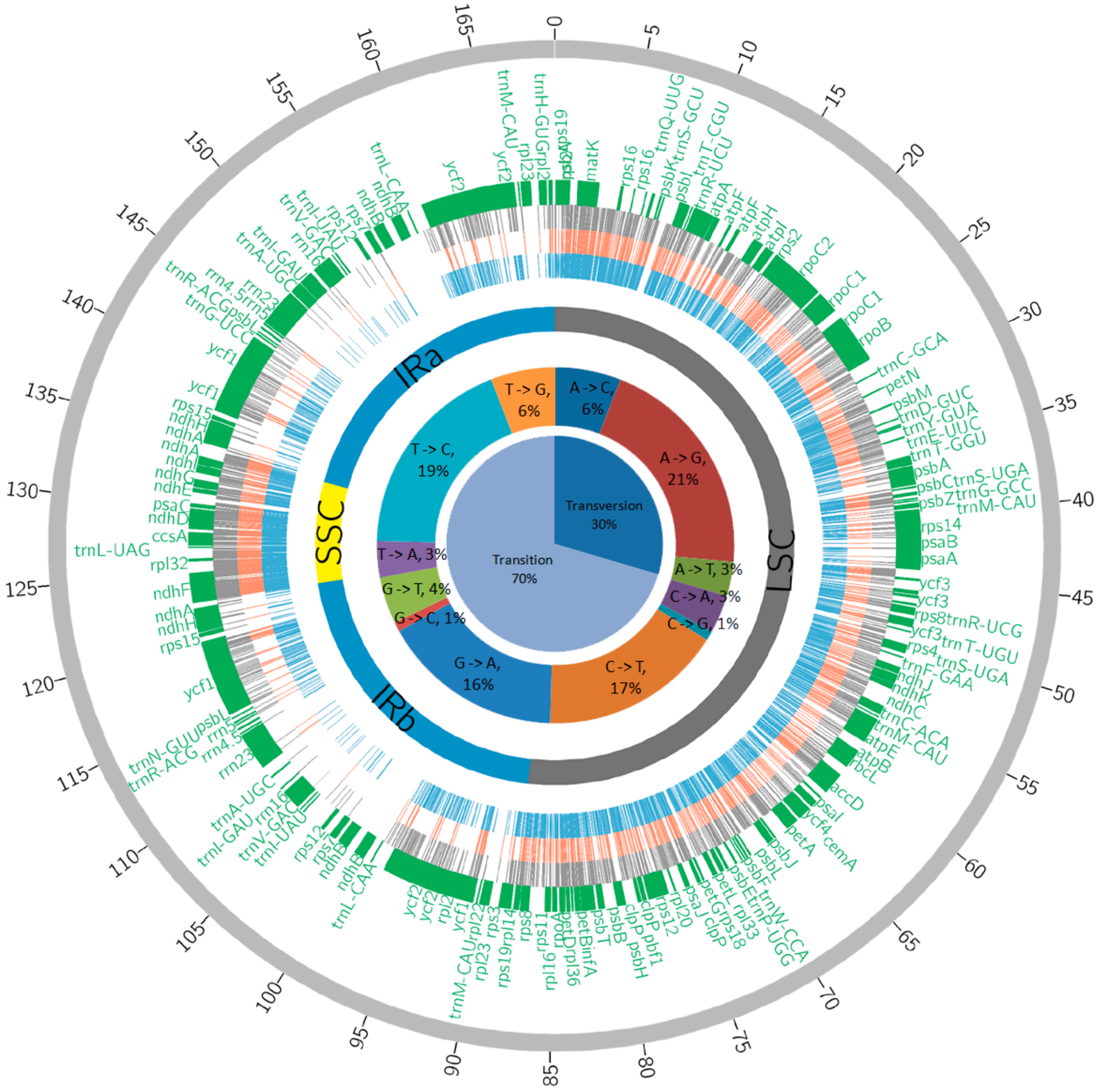

Figure S5. CP Genome wide SNP distribution Ensete ventricosum.

a

b

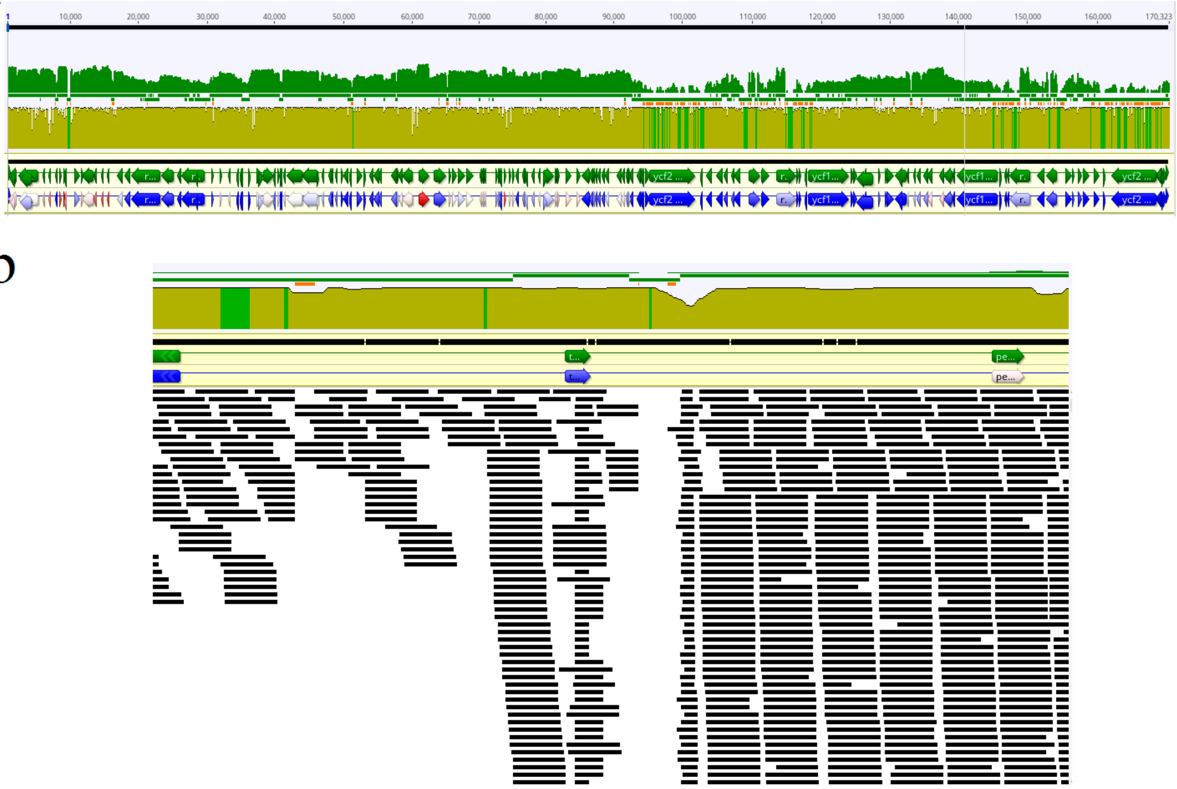

Figure S6. Mapping of leaf transcriptome on the reference CP sequences genome.
